# The nutritional composition of the vegetable soybean (maodou) and its potential in combatting malnutrition

**DOI:** 10.3389/fnut.2022.1034115

**Published:** 2023-01-05

**Authors:** Kwadwo Gyapong Agyenim-Boateng, Shengrui Zhang, Shibi Zhang, Aimal Nawaz Khattak, Abdulwahab Shaibu, Ahmed M. Abdelghany, Jie Qi, Muhammad Azam, Caiyou Ma, Yue Feng, Huoyi Feng, Yitian Liu, Jing Li, Bin Li, Junming Sun

**Affiliations:** The National Engineering Research Center for Crop Molecular Breeding, MARA Key Laboratory of Soybean Biology (Beijing), Institute of Crop Sciences, Chinese Academy of Agricultural Sciences, Beijing, China

**Keywords:** soybean (*Glycine max* L. Merrill), maodou, macronutrients, micronutrients, malnutrition

## Abstract

**Introduction:**

Global malnutrition continues to be a canker owing to poor eating habits and over-reliance on the major staple crops. Vegetable soybean (maodou) is gaining popularity globally as an affordable snack and vegetable.

**Methods:**

In this study, we profiled the nutritional composition of 12 soybean cultivars at the vegetable (R6-R7) and mature (R8) stages. We also conducted an RNA-seq analysis during seed development, focusing on key biosynthesis enzymes for quality traits.

**Results:**

The results showed that 100 g of maodou contained 66.54% moisture, 13.49% protein, 7.81% fatty acids, 2.47% soluble sugar, abundant content of minerals, and micronutrients, including folate (462.27 μg FW) and carotenoids (3,935.41 μg FW). Also, the isoflavone content of maodou ranged between 129.26 and 2,359.35 μg/g FW. With regard to the recommended daily allowance, 100 g fresh weight of maodou can contribute 26.98, 115.57, and 11.60% of protein, folate, and zinc, respectively, and significant proportions of other nutrients including linoleic acid (21.16%), linolenic acid (42.96%), zinc (11.60%), and iron (18.01%). On a dry weight basis, maodou has two to six folds higher contents of folate, tocopherol, and carotenoid than the mature soybean. Furthermore, RNA-seq analysis revealed that key biosynthesis enzymes of quality traits are differentially expressed during seed development and may contribute to variations in the content of quality traits at the vegetable and mature stages. Correlation analysis of quality traits at both stages revealed that protein only correlated positively with zinc at the vegetable stage but negatively correlated with total tocopherol and total fatty acid at the mature stage. Complex associations among folates, soluble sugar, and isoflavones were also identified.

**Discussion:**

This study provides insight into the nutritional contents of vegetable soybean and demonstrates that maodou is essential for meeting the nutritional requirements of most countries.

## Graphical Abstract

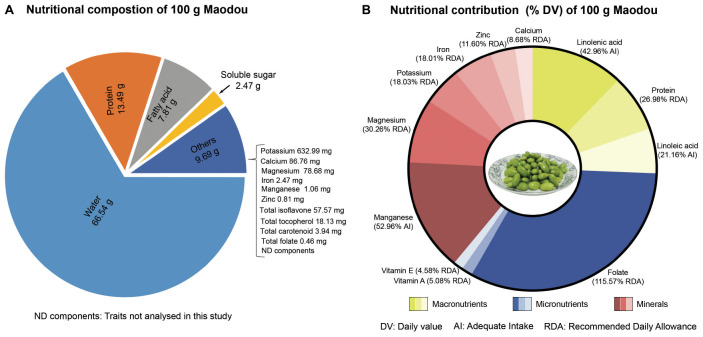

## 1. Introduction

Malnutrition refers to the deficiencies, excesses, and or imbalances in a person’s intake of energy and or nutrients (1). Different forms of malnutrition exist, including undernutrition, overweight, and micronutrient malnutrition, which are specific to different environments and are caused by different factors. For instance, in developing countries, undernutrition and micronutrient malnutrition rates are high, which is attributable to inadequate caloric or protein intake and the consumption of lower amounts of micronutrients (2). On the other hand, overweight and micronutrient deficiencies are rampant in developed countries due to the excessive consumption of calories and insufficient essential micronutrients (3). In most studies, it is revealed that the over-reliance on the three major crops (wheat, maize, and rice) and inappropriate dietary choices may be the highest contributing factors to malnutrition (4), and researchers suggest that complementing these staple crops with leguminous crops may increase the overall nutritional uptake and balance (5).

Legumes are an essential part of the diet of most parts of the world not only because they are rich in macro and micronutrients but also because of their low production costs and adaptability in many environments. Among legumes, soybean has been the most cultivated in the past three decades (6). It is an important source of protein and oil for human beings, livestock, and poultry and provides the raw material for bioenergy, pharmaceutical, and technical industries. Furthermore, soybean can be grown throughout the year in the tropics and subtropics with simple agronomic practices and can also improve the soil by spontaneously fixing nitrogen into the soil. Soybean protein is one of the least expensive sources of dietary proteins and is considered a good substitute for animal protein as soybean protein contains all the essential amino acids, including lysine, which is especially low in other staple crops. Also, soybean oil is widely preferred due to its high polyunsaturated and low saturated fatty acid content. Besides protein and oil, soybean is also rich in a spectrum of highly essential nutrients, including bioactive components, phytonutrients, and minerals. Thus, the soybean crop has been an essential part of Asian cuisine for thousands of years.

The soybean crop can be transformed into many food types and consumed at different maturation stages, including the sprouting, vegetable, and mature stages (7). Mature soybean, which is widely known, is harvested dry and processed into soy-based foods (tofu, soy sauce, soybean milk, natto, and many others). On the other hand, vegetable soybeans, known as “*Maodou* (毛豆)” in China and “*Edamam*e” in Japan, are harvested at the immature stage (R6–R7 stage) when the seed fills 80–90% of the pod cavity (8). The mature soybean seed is mainly processed before consumption, which may result in the loss of nutrients. Meanwhile, vegetable soybean is consumed as a whole seed, either as a snack or a vegetable. As a snack, the pods of maodou are cooked lightly in salty water for a few minutes (see Supplementary Video), and as a vegetable, the seeds can be fried with meat or other vegetables or cooked shortly in boiled water for salad. Thus, shorter cooking times and consumption of the whole seed for maodou may significantly limit the nutritional losses, suggesting the importance of maodou, if incorporated into our diets. In recent years, consumption of maodou has gained popularity in more western countries, especially in USA, due to its high nutritional value.

As such, studies have highlighted maodou as a significant source of plant-based proteins. Despite this, maodou production constitutes less than 2% of global soybean production, and the nutritional profile of the maodou has not been well studied compared to the mature soybean (9). To exploit the full potential of maodou, its nutritional composition must be well known. In general, the nutritional content of maodou could have immense benefits in food industries, assist in biofortification studies and eventually contribute to the fight against malnutrition. To address this, we profiled the nutritional content of maodou, including folates, carotenoids, tocopherols, and minerals. Further, we evaluated the nutritional contribution of the consumption of 100 g of maodou per day and analyzed the expression of key biosynthesis enzymes for soybean quality traits during seed development.

## 2. Materials and methods

### 2.1. Plant materials

**FIGURE 1:**
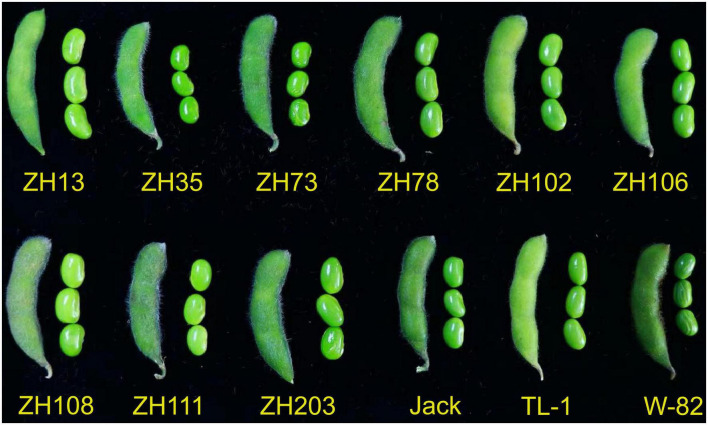
Soybean cultivars used in the study at the maodou stage.

Twelve soybean cultivars, namely; Zhonghuang 13 (ZH13, high protein content), Zhonghuang 35 (ZH35, high oil content), Zhonghuang 73 (ZH73, high-yielding), Zhonghuang 78 (ZH78, high oil content and low off-flavor), Zhonghuang 102 (ZH102, low off-flavor), Zhonghuang 106 (ZH106, high oil content), Zhonghuang 108 (ZH108, high-yielding), Zhonghuang 111 (ZH111, high-yielding), Zhonghuang 203 (ZH203, high oil, isoflavone contents, and low off-flavor), Tianlong 1 (TL-1), Jack and William 82 (W-82) were used in this study (Figure 1 and Supplementary Table 1). All plants were grown in Changping (40^°^ 13′ N and 116^°^ 12′ E), Beijing, China, in the summer of 2021. The seeds of each cultivar were planted in 3.00 m long rows with 0.10 and 0.50 m intra and inter-row spacing. Recommended agronomic practices were followed. The soybean cultivars were harvested at the immature (R6–R7) and the dry mature stages (R8). Immature soybeans were freeze-dried and stored at −80°C until use. To quantify quality traits, seeds were finely ground using mortar and pestle and stored at −20°C until analysis. However, for protein and mineral analysis, samples were dried for 48 h at 65°C before analysis. Extraction was done in triplicate for each trait. Immature or vegetable soybeans will hereafter be referred to as maodou in the manuscript for uniformity.

### 2.2. Physical properties and moisture content of soybean pods and seeds

Pod length, width, and thickness were measured using a digital fractional caliper. Pod and seed weight were measured on a Sartorius AG scale (Sartorius Lab Instruments Gmbh & Co. KG, Germany). The moisture content of soybean samples was determined in triplicate by incubating samples at 65°C for 48 h using an electric oven (10).

### 2.3. Extraction and quantification of protein, fatty acids, and soluble sugars

Crude protein content was determined by the Kjeldahl method to obtain the total nitrogen content, which was then multiplied by a protein conversion factor of 6.25 (11). Fatty acids were extracted by weighing 30 mg of soybean powder into screw cap tubes containing 1 ml of 2.50% H_2_SO_4_. Samples were heated at 85°C for 1 h 30 min, with shaking at every 10-min interval, and were cooled for 10 min. Further, 150 μL of NaCl and 700 μL of Hexane were added, after which samples were shaken for 3 min. Samples were centrifuged at 4000 rpm for 10 min, and the supernatant was taken into 2 ml vials for gas chromatography analysis. Soluble sugars were determined as described by Azam et al. (12). Briefly, 100 mg of soybean powder was weighed and mixed with 50% acetonitrile. Samples were shaken for 8 h at room temperature in an incubator. Five hundred milliliters of the supernatant were transferred into a new tube containing 200 μL acetonitrile. The mixture was shaken to precipitate the protein and was left at room temperature for 10 min. The samples were centrifuged at 20°C for 10 min, and the supernatant was filtered using syringe filters (0.22 μm) before detection using UPLC-RID.

### 2.4. Folate, tocopherol, carotenoid, and isoflavone extraction and quantification

Folate monoglutamates, including tetrahydrofolate (THF), 5-methyltetrahydrofolate (5MTHF), 5,10-methenyltetrahydrofolate (5,10MTHF), 10-formylfolic acid (10FFA), 5-formyltetrahydrofolate (5FTHF), dihydrofolate (DHF) and folic acid (FA), were extracted and quantified using an HPLC-MS/MS system described previously (13). Carotenoids, including beta-carotene (β-carotene), alpha-carotene (α-carotene), lutein, zeaxanthin, and beta-cryptoxanthin (β-cryptoxanthin) were analyzed as described by Gebregziabher et al. (14). Tocopherols, including delta-tocopherol (δ-tocopherol), gamma-tocopherol (γ-tocopherol), and alpha-tocopherol (α-tocopherol), were extracted and quantified as described by Ghosh et al. (15). Isoflavone was determined as described by Sun et al. (16) with minor modifications. For isoflavone extraction, 20 mg of soybean powder was weighed into a buffer (70% ethanol and 0.10% acetic acid). Samples were shaken for 12 h in a shaker incubator and were centrifuged at 6,000 rpm for 10 min. The supernatant was filtered using syringe filters (0.22 μm) and detected using a UPLC system (Waters Corp.). The separation was performed by ACQUITY UPLC BEH C18 column (1.7 μm, 1.0 mm × 50 mm C18, Waters Corp.) using acetonitrile (*v/v* 0.10% formic acid) and water (*v/v* 0.10% formic acid) as solvents A and B, respectively. A linear gradient profile from 12.50 to 18.50% A from start to 2 min, stayed at 18.50% A for 3 min, up to 27% A to 9 min, up to 30% A to 12 min, was decreased to 12.50% A for 1 min and was allowed to equilibrate for 2 min before the next run. Detection was carried out using a UV detector with a wavelength of 254 nm.

### 2.5. Extraction and quantification of mineral content

The extraction of minerals was performed by weighing 0.10 g of dried soybean powder mixed with 4 ml of concentrated nitric acid. The mixture was digested using a super microwave digestion instrument (Hangzhou Puyu Company). After dilution, the sample was tested using ICP-MS (Hangzhou Puyu Company). The minerals detected were magnesium (Mg), potassium (K), calcium (Ca), manganese (Mn), iron (Fe), and zinc (Zn).

### 2.6. RNA-seq-based gene expression of key enzymes for quality traits during seed development

RNA-seq analysis was conducted for ZH13, ZH35, ZH73, and ZH78 during seed development. Samples of each cultivar were taken every week, starting from the R5 stage (rapid nutrient redistribution from the plant to the seed) to the mature stage. In all, samples were taken from seven time-points. At every time-point, three biological replicates (at least 5 pods per plant) were taken for each cultivar. Seeds were then finely ground, and plant RNA was extracted using the RNA Easy Fast Plant Tissue Kit (Tiangen Biotech, Beijing, China) for RNA-seq. Agilent and Nanodrop instruments were used to determine the integrity and purity of the RNA, respectively. RNA-seq analysis was conducted by the BLgene company limited (Beijing, China) with three technical replicates for each sample. Raw RNA-seq datasets were aligned with the Williams 82 a2 v275 reference genome^1^ using HISAT2 (17). Read numbers for each gene were counted using featureCounts (18) based on a gene transfer format (GTF)-formatted file generated by Cufflinks (19). Transcripts per million (TPM) values of the assembled transcription units were calculated and normalized using DESeq2 (20) with global normalization parameters.

### 2.7. Statistical analysis

All data are from triplicate measurements and were subjected to analysis of variance (ANOVA) using the *Agricolae* package^2^ in R 3.4.5 (R Foundation for Statistical Computing, Vienna, Austria). *Post hoc* mean separation was done using Tukey’s HSD at *P* < 0.05. Scatter plots were produced using GraphPad Prism version 9.00 for Windows. For hierarchical clustering, data for all treatments were normalized to a scale of 0–1 and were transferred to Tbtools (version 1.098691) to generate the hierarchical clustering and heatmap of the 12 soybean seeds, maturation stage, and quality traits. Principal component analysis (PCA) was performed in R using the *FactoMineR* package^3^ to evaluate the segregation of the quality traits based on the stage. Total tocopherol content was estimated as the sum of tocopherol isomers delta, gamma, and alpha-tocopherol. Total carotenoid was calculated as the sum of all individual carotenoids (β-carotene, α-carotene, lutein, zeaxanthin, and β-cryptoxanthin). Total isoflavone content was calculated as the sum of individual isoflavones (daidzin, glycitin, genistin, malonyldaidzin, malonylglycitin, and malonylgenistin). Total folate content was estimated as the sum of individual folate vitamers (THF, 5MTHF, 5,10MTHF, 10FFA, 5FTHF, DHF, and FA). Total soluble sugar content was calculated as the sum of fructose, glucose, raffinose, stachyose, and sucrose. Total fatty acid was calculated as the sum of individual components, palmitic acid, stearic acid, oleic acid, linoleic acid, and linolenic acid. All quality traits were expressed on fresh weight (FW) and dry weight (DW) basis.

## 3. Results

### 3.1. Physical properties and moisture content of soybean pods and seeds

Physical properties, including seed size, pod weight, pod length, and pod thickness, are desirable properties that determine the acceptability of vegetable soybean and can assist in genotype selection for breeding projects. The physical properties and moisture content of cultivars evaluated in this study are shown in Table 1. In this study, the seed weight of maodou varied between 20.65 and 59.15 g per 100-seed. The average hundred-seed weight was 45.20 g, which was in agreement with a previous study by Xu et al. (21), with the highest hundred seed weight (59.15 g) observed in ZH78, followed by ZH13 at 58.15 g. Consistent with seed size, pod weight ranged from 1.36 to 3.12 g, with the highest in ZH78 and ZH13.

**TABLE 1:** Physical properties and moisture content of soybean pods and seeds at the vegetable and mature stages.

Cultivar	Stage	100-seed weight (g)	Moisture content (%)	Pod length (mm)	Pod width (mm)	Pod thickness (mm)	Pod weight (g)
ZH 13	Vegetable	58.15 ± 1.05^a^	63.92 ± 2.69^a^	57.69 ± 3.38^a^	12.23 ± 0.45^a^	9.31 ± 0.27^a^	3.03 ± 0.13^ab^
	Mature	26.40 ± 0.78^b^	6.39 ± 0.27^b^				
ZH 35	Vegetable	20.65 ± 0.20^a^	72.90 ± 1.57^a^	49.10 ± 1.88^bcd^	9.50 ± 0.73^d^	6.09 ± 0.18^d^	1.36 ± 0.02^d^
	Mature	15.97 ± 0.25^b^	6.35 ± 0.03^b^				
ZH 73	Vegetable	30.10 ± 0.18^a^	70.83 ± 0.82^a^	43.98 ± 1.78^d^	10.11 ± 0.73^cd^	7.04 ± 0.59^cd^	1.78 ± 0.25^cd^
	Mature	20.18 ± 0.37^b^	6.53 ± 1.29^b^				
ZH 78	Vegetable	59.15 ± 0.28^a^	67.13 ± 1.64^a^	55.43 ± 2.57^ab^	11.25 ± 0.50^abc^	9.03 ± 0.80^ab^	3.12 ± 0.72^a^
	Mature	25.17 ± 1.23^b^	6.65 ± 0.65^b^				
ZH 102	Vegetable	44.60 ± 0.74^a^	64.14 ± 5.35^a^	58.62 ± 1.99	11.63 ± 0.31^ab^	8.49 ± 0.31^ab^	2.28 ± 0.26^abcd^
	Mature	23.70 ± 0.66^b^	7.05 ± 0.52^b^				
ZH 106	Vegetable	49.38 ± 0.40^a^	65.60 ± 1.78^a^	52.46 ± 1.98	11.12 ± 0.65^abc^	9.02 ± 0.50^ab^	1.94 ± 0.18^bcd^
	Mature	16.18 ± 0.75^b^	6.55 ± 0.34^b^				
ZH 108	Vegetable	48.93 ± 0.31^a^	64.57 ± 2.32^a^	56.57 ± 2.95	12.25 ± 0.72^a^	9.25 ± 0.39^ab^	2.58 ± 0.19^abc^
	Mature	20.65 ± 1.10^b^	6.66 ± 0.10^b^				
ZH 111	Vegetable	44.39 ± 0.43^a^	66.21 ± 5.15^a^	51.65 ± 2.26	11.3 ± 0.18^abc^	8.58 ± 0.12^ab^	2.00 ± 0.06^bcd^
	Mature	19.37 ± 1.06^b^	7.13 ± 0.20^b^				
ZH 203	Vegetable	53.03 ± 0.14^a^	66.51 ± 4.13^a^	57.65 ± 0.51	11.68 ± 0.12^ab^	8.47 ± 0.27^ab^	2.85 ± 0.31^abc^
	Mature	24.03 ± 0.94^b^	6.71 ± 0.22^b^				
Jack	Vegetable	44.28 ± 0.46^a^	67.35 ± 0.24^a^	46.33 ± 3.23	10.50 ± 0.26^bcd^	8.89 ± 0.18^ab^	1.96 ± 0.18^bcd^
	Mature	15.52 ± 0.38^b^	6.75 ± 0.47^b^				
TL-1	Vegetable	44.69 ± 0.23^a^	62.19 ± 3.96^a^	55.95 ± 2.72	10.93 ± 0.06^abc^	8.10 ± 0.17^bc^	2.20 ± 0.17^abcd^
	Mature	18.92 ± 0.91^b^	7.19 ± 0.79^b^				
W-82	Vegetable	44.99 ± 1.00^a^	67.08 ± 2.50^a^	53.28 ± 2.75	11.37 ± 0.33^abc^	8.73 ± 0.31^ab^	2.52 ± 0.23^abc^
	Mature	19.20 ± 0.05^b^	6.59 ± 0.67^b^				

Different lowercase letters indicate statistical differences at *P* < 0.05. For 100-seed weight and moisture content, comparisons were made between two stages for each cultivar. Pod traits were only analyzed at the vegetable stage with comparisons made among cultivars.

This study reveals that both seed and pod weight can be used to determine the size of the vegetable soybean. Large seeded maodou genotypes are preferred and attractive to customers. The large-seeded cultivars in this study can be further studied and incorporated into future breeding programs to develop superior vegetable-type soybeans. The average pod width was 11.16 mm, while the average pod thickness was 8.42 mm. Also, the pod length of the maodou ranged from 43.98 to 58.62 mm (Table 1).

Moisture content affects the organoleptic value, which is a determiner of the maturity and marketability of maodou. Studies indicate that soybean moisture declines with maturity, and either extremely high or low moisture contents indicate that the soybean has already matured or is yet to mature and, as such, cannot be classified as vegetable soybean. Maodou seeds had moisture content ranging from 62.19 to 72.90%, with an average of 66.54% (Table 1).

Furthermore, the seed weight and moisture content of maodou and mature soybean seeds were compared. Seed weight decreased significantly at the mature stage, with the mean seed weight of the mature soybean cultivars (20.44 g) almost half that of maodou (45.19 g) (Table 1).

### 3.2. Macronutrient composition of maodou

**FIGURE 2:**
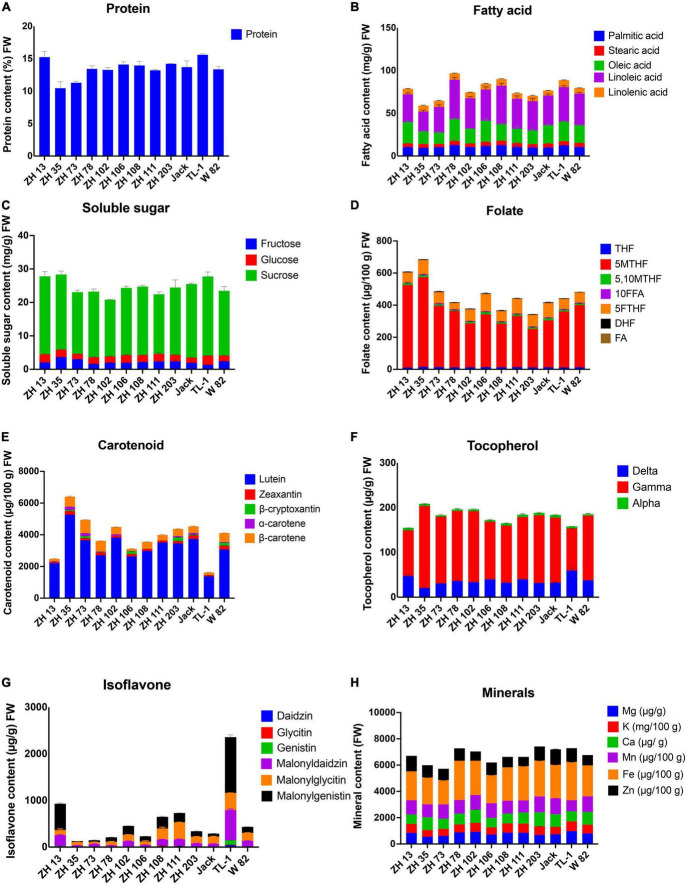
Nutritional composition of maodou (FW). **(A)** Protein, **(B)** fatty acids, **(C)** soluble sugars, **(D)** folates, **(E)** carotenoids, **(F)** tocopherols, **(G)** isoflavones, and **(H)** minerals.

Macronutrients are needed in large amounts in humans, provide energy, maintain fundamental body structure, help prevent diseases and allow normal functioning of the body. Our study showed that maodou is a rich source of proteins. In this study, the mean protein content of maodou on a fresh weight basis among cultivars was 13.49% (40.28% DW), ranging from 10.46% (ZH35) to 15.60% (TL-1) (Figure 2A and Supplementary Table 2).

Concerning the fatty acid profile of maodou, all five major fatty acids: palmitic acid, stearic acid, oleic acid, linoleic acid, and linolenic acid, were detected. Total fatty acid contents ranged from 5.90 to 9.67 g/100 g FW (average; 7.81 g/100 g FW), with ZH78 containing the highest amount of total fatty acids. Palmitic acid, stearic acid, oleic acid, linoleic acid, and linolenic acid contents varied from 0.93 to 1.25 g/100 g FW, 0.39 to 0.53 g/100 g FW, 1.35 to 2.56 g/100 g FW, 2.35 to 4.62 g/100 g FW, and 0.58 to 0.81 g/100 g FW, respectively (Figure 2B and Supplementary Table 2).

The soluble sugar profile of maodou included fructose, glucose, and sucrose. The average total soluble sugar content was 2.47 g/100 g, whereas fructose, glucose, and sucrose contained average values of 0.22 g/100 g FW, 0.21 g/100 g FW, and 2.04 g/100 g FW, respectively (Figure 2C and Supplementary Table 2). ZH35 had the highest total soluble sugar (2.83 g/100 g FW) and fructose (0.36 g/100 g FW) contents; TL-1 had the highest sucrose content of 2.37 g/100 g.

### 3.3. Composition of micronutrients and isoflavones in maodou

Micronutrients are needed in lesser amounts but play a significant role in the body’s metabolic activities. They are usually acquired from external sources, with the most important source being food. The micronutrients studied here include folate monoglutamates, tocopherol, and carotenoid (Supplementary Table 4). Seven folate monoglutamate vitamers, THF, 5MTHF, 5,10MTHF, 10FFA, 5FTHF, DHF, and FA, were detected in maodou (Figure 2D). The mean total folate content of soybean was 462.27 μg/100 g FW. The average THF content at the vegetable stage was 12.55 μg/100 g FW, whereas the mean content of 5MTHF was 356.18 μg/100 g FW. The mean 5FTHF content of maodou was 75.07 μg/100 g FW. The minor components, 5,10MTHF and DHF, ranged from 5.66 to 14.27 μg/100 g FW and 1.61 to 3.60 μg/100 g FW, respectively. The average 10FFA and FA contents were 4.33 μg/100 g FW and 1.00 μg/100 g FW, respectively. The highest 5MTHF and total folate contents were observed in ZH35 at 558.71 μg/100 g and 685.81 μg/100 g, respectively.

For carotenoids, total carotenoid content was 3935.41 μg/100 g FW in maodou. Also, β-carotene ranged from 150.00 to 847.87 μg/100 g FW, α-carotene varied from 7.59 to 183.77 μg/100 g FW, β-cryptoxanthin ranged from 22.56 to 229.83 μg/100 g FW, zeaxanthin ranged from 63.54 to 250.73 μg/100 g FW, and lutein content was from 1376.91 to 5259.64 μg/100 g FW (Figure 2E and Supplementary Table 4). The most dominant carotenoid at the vegetable stage was lutein, whereas β-cryptoxanthin and α-carotene were not detected in some cultivars. In maodou, ZH35 contained the highest amounts of total carotenoids (6410.35 μg/100 g FW), lutein (5259.64 μg/100 g FW), and α-carotene (183.77 μg/100 g FW), whereas the highest amounts of β-carotene, zeaxanthin and β-cryptoxanthin were observed in ZH73 (847.87 μg/100 g FW), W-82 (250.73 μg/100 g FW), and ZH203 (178.71 μg/100 g FW), respectively.

The average total tocopherol content of maodou was 181.27 μg/g FW, with the most dominant tocopherol isomer being γ-tocopherol at an average of 140.10 μg/g FW, followed by δ-tocopherol and α-tocopherol at mean concentrations of 36.77 and 4.58 μg/g FW, respectively (Figure 2F). The highest total tocopherol content was seen in ZH35 (208.75 μg/g FW) and the lowest in ZH13 (154.95 μg/g FW).

The isoflavone contents of soybean cultivars are shown in Figure 2G and Supplementary Table 6. Total isoflavone content varied from 129.26 to 2359.35 μg/g FW in maodou. Average malonyldaidzin, malonylglycitin, and malonylgenistin contents of maodou were 143.63, 164.89, and 248.58 μg/g FW, respectively. Daidzin, glycitin, and genistin were detected in a few cultivars at the vegetable stage. Total isoflavone content was the highest in TL-1, followed by ZH13 and ZH111.

### 3.4. Mineral contents of maodou

Minerals are essential nutrients vital for humans. In this study, we profiled the magnesium, potassium, calcium, manganese, iron, and zinc contents of maodou (Figure 2H and Supplementary Table 8). The average magnesium content for maodou was 78.68 mg/100 g FW. At an average of 632.99 mg/100 g FW, the potassium content of maodou varied between 501.36 and 743.67 mg/100 g FW. Maodou contained an average of 86.76 mg/100 g FW, 1.06 mg/100 g FW, and 2.47 mg/100 g FW of calcium, manganese, and iron, respectively. Meanwhile, the average zinc content in maodou was 0.81 mg/100 g FW. In maodou, magnesium and potassium contents were the highest in TL-1, calcium was the highest in ZH203, manganese was the highest in Jack, iron was the highest in ZH78, and zinc was the highest in ZH13.

### 3.5. Variations in quality traits in vegetable and mature soybean cultivars

**FIGURE 3:**
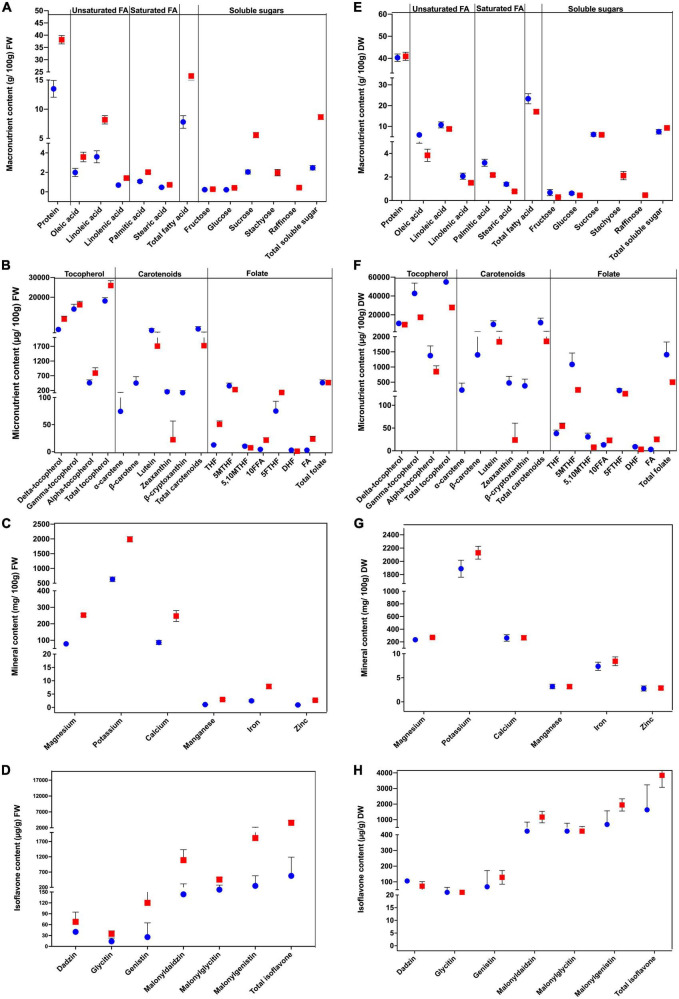
Variations in the nutritional composition of maodou (blue) and mature soybean (red). **(A)** Macronutrient, **(B)** micronutrient, **(C)** mineral, and **(D)** isoflavone contents calculated on a fresh weight basis (FW) (left panel). **(E)** Macronutrient, **(F)** micronutrient, **(G)** mineral, and **(H)** isoflavone contents calculated on a dry weight basis (DW) (right panel).

We evaluated the differences in the nutritional content of the soybean cultivars at both vegetable and mature stages, and the results are shown in Figure 3. The average moisture content of maodou was 66.54%, whereas the average moisture content of mature soybean was 6.71%. Moisture content will affect differences in quality traits. Hence, to clearly understand the differences between the studied traits at both stages, the contents of quality traits were calculated on dry weight basis, where moisture content is assumed to be zero (Figure 3 and Supplementary Tables 10–18). There was no significant difference in the protein content at both stages (*P* > 0.05). The average protein content of maodou was 40.24 g/100 g and mature soybean contained 40.90 g/100 g of protein. Total fatty acid content was 23.32 g/100 g in maodou and 18.93 g/100 g in mature soybean (*P* < 0.05). Consistently, all individual fatty acid components were significantly higher in maodou than in mature soybean. On the other hand, total soluble sugar content was significantly higher in mature soybean (9.27 g/100 g) than maodou (7.44 g/100 g), which may be partly because the two oligosaccharides (stachyose and raffinose) were not detected in maodou. However, fructose and glucose contents were significantly higher in maodou. Contrastingly, no significant difference was observed between the sucrose content of maodou and mature soybean.

The average total folate content was 1401.06 μg/100 g and 500.86 μg/100 g for maodou and mature soybean, respectively. Significant differences were observed for all folate vitamers between both stages. The average THF content was 38.06 μg/100 g in maodou, lower than the content in the mature soybean (54.31 μg/100 g). Similarly, FA content, which was 3.07 μg/100 g in maodou increased exponentially to 25.09 μg/100 g in mature soybean. In like manner, 10FFA content was higher by 0.57-fold in the mature soybean than maodou. On the contrary, the average 5MTHF content in maodou (1085.34 μg/100 g) was 4.44-fold more than the mature soybean (245.93 μg/100 g). Average 5,10MTHF was also 3.94-fold higher in maodou than the mature soybean. The average 5FTHF content was 227.10 μg/100 g in maodou but was decreased in the mature stage at 141.69 μg/100 g. Regarding carotenoids, total carotenoid content was significantly higher in maodou (12130.75 μg/100 g) than in mature soybean (1840.31 μg/100 g). Particularly, α-carotene, β-carotene and β-cryptoxanthin were exclusively detected in maodou. Zeaxanthin was hardly detected in the mature soybean at an average of 23.73 μg/100 g, but was highly present in maodou at 476.36 μg/100 g. Lutein content was 5.3-fold higher in maodou (9830.66 μg/100 g) than mature soybean (1830.42 μg/100 g). Total tocopherol content for maodou was 548.97 μg/g, whiles mature soybean was 278.09 μg/g. γ-tocopherol and α-tocopherol contents were also significantly higher in maodou than the mature soybean. However, no significant difference was seen for the δ-tocopherol content at both stages.

The magnesium, potassium, and iron contents differed significantly between the two stages with the higher contents seen in the mature soybean. In the mature soybean, the average magnesium, potassium contents were 270.88 mg/100 g, 2129.81 mg/100 g, and 8.43 mg/100 g, respectively. In maodou, the average magnesium, potassium and iron contents were 234.05 mg/100 g, 1889.16 mg/100 g, and 7.38 mg/100 g, respectively. For calcium, manganese, and zinc, no significant differences were seen between the two stages. With the exception of malonylglycitin content, all other isoflavone components and total isoflavone differed significantly between the two stages. Total isoflavone content was 1633.55 μg/g in maodou but was double in mature soybean at 3841.81 μg/g. Components including daidzin and glycitin were significantly higher in maodou by 1.46 and 1.05-fold. Meanwhile, genistin, malonyldaidzin and malonylgenistin contents were higher in the mature soybean by 0.53, 0.35, and 0.36-fold, respectively.

### 3.6. Expression of key enzymes involved in quality traits based on RNA-seq analysis

**FIGURE 4:**
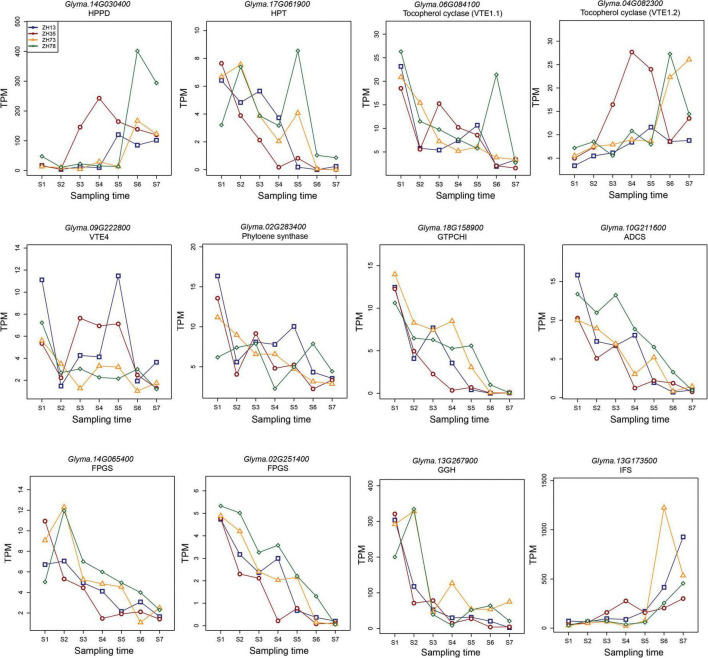
Expression of key biosynthesis enzymes for tocopherol, carotenoid, folate, and isoflavone during seed development in soybean. Sampling times, S1–S7 represent the different sampling time points in sequential order and S1 corresponds to the R5 stage of soybean seed development, while S7 corresponds to the mature stage.

Key enzymes play specific roles in the accumulation of quality traits. In most instances, their expression levels at specific stages may indicate the accumulation of the specific compounds they synthesize. To elucidate the differences in the contents of tocopherol, carotenoid, folate, and isoflavone at the maodou and mature stage, we studied the expression of key biosynthesis enzymes of these traits in soybean during seed development using RNA-seq analysis (Figure 4). This will be a guideline for breeders to facilitate molecular breeding of elite varieties which will be useful for industries and consumers. For this reason, seed samples were taken weekly from the R5 stage. Soybean genes are mostly comprised of multiple isoforms. In this study, gene isoforms with higher relative expression levels are shown in the manuscript (Figure 4), and other isoforms with lower expressions are described in the Supplementary material (Supplementary Table 19).

Homogentisate phytyltransferase (HPT), *p*-Hydroxyphenylpyruvate dioxygenase (HPPD), gamma-tocopherol methyltransferase (VTE4), and tocopherol cyclase (VTE1) are the major enzymes in the tocopherol pathway. Genetic engineering studies have frequently targeted these enzymes to develop transgenic plant lines with increased tocopherol contents (22). In our study, the relative expression of *GmHPPD* was relatively low from S1 to S5 but increased significantly in the S6 and S7 stages. On the other hand, the relative expressions of *GmHPT* and *GmVTE4* decreased with maturity, with the highest expression seen at the early stages. VTE consisted of two isoforms, *GmVTE1.1* and *GmVTE1.2*, which exhibited contrasting expressions during seed development. *GmVTE1.1* expression levels reduced with maturity, and *GmVTE1.2* increased with maturity.

Phytoene synthase (PSY) catalyzes the first committed step in the carotenoid biosynthesis pathway and is a major rate-limiting enzyme of carotenogenesis (23). In this study, we identified four isoforms of PSY in soybean. Consistently, all isoforms were highly expressed in the S1 stage but steadily reduced with maturity. *GmPSYs* were the least expressed at the S7 stage.

Folate biosynthesis in plants is composed of 11 enzymes. Out of these, four enzymes have been reported to be key enzymes in folate biosynthesis. They have been engineered in multiple studies to determine their effect on folates in plants (24–26). These enzymes include GTP cyclohydrolase I (GTPCHI), aminodeoxychorismate synthase (ADCS), folylpolyglutamate synthetase (FPGS), and gamma-glutamyl hydrolase (GGH). In this study, soybean gene *GmGTPCH1*, which consisted of two isoforms, was highly expressed in the early stages of seed development, but its expression declined with maturity. Similarly, *GmADCS* also consisted of two isoforms and declined with maturity. Three isoforms of *GmFPGS* were identified, and these 3 isoforms steadily declined with maturity. Consistent with other folate enzymes, the two isoforms of *GmGGH* reduced steadily with maturity, with the highest expressions in the S1 and S2 stages.

2-hydroxy-isoflavone synthase (isoflavone synthase or IFS) is a crucial enzyme that differentiates isoflavone-producing plants from non-isoflavone-producing plants (27). In soybean, we identified two isoforms of IFS, *GmIFS1*, and *GmIFS2*, whose expressions were the same through seed development. The expression of IFS was relatively low from S1 to S5 but increased exponentially in the S6 and S7 stages.

### 3.7. Correlation of quality traits at the vegetable and mature stages

**FIGURE 5:**
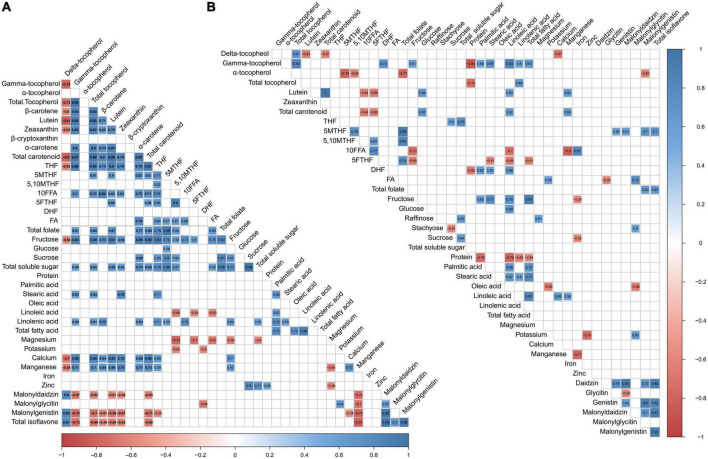
Correlation analysis of quality traits at vegetable and mature stages. **(A)** Vegetable stage and **(B)** mature stage. Empty cells indicate no significant correlations between traits (*P* > 0.05).

In this study, we analyzed the correlation between all quality traits at both the vegetable and mature stages to identify their associations (Figure 5). In maodou, protein only correlated positively with zinc, however, negatively correlated with γ-tocopherol, total tocopherol, DHF, linoleic acid, linolenic acid and total fatty acid in the mature soybean. Total soluble sugar positively correlated with four folate monoglutamates and total folate in maodou but only positively correlated with THF in mature soybean. Total fatty acid was no correlation with other traits in maodou but negatively correlated with protein and 5FTHF in mature soybean. Isoflavones, positively correlated with δ-tocopherol, but negatively correlated with total tocopherol and total carotenoid in maodou. However, in mature soybean, isoflavone positively correlated with 5MTHF and total folate. Total tocopherol positively correlated with total soluble sugar in maodou but no correlation was observed between them in the mature soybean. Total carotenoid positively correlated with total folate, total soluble sugar and negatively correlated with δ-tocopherol in maodou. Moreover, a consistent negative correlation was also observed between total carotenoid and δ-tocopherol in mature soybean. However, no association between total carotenoid, folate or soluble sugar was observed in mature soybean. Despite having no significant associations with isoflavone in maodou, total folate positively correlated with total isoflavone in the mature soybean.

### 3.8. Hierarchical cluster analysis and principal component analysis

**FIGURE 6:**
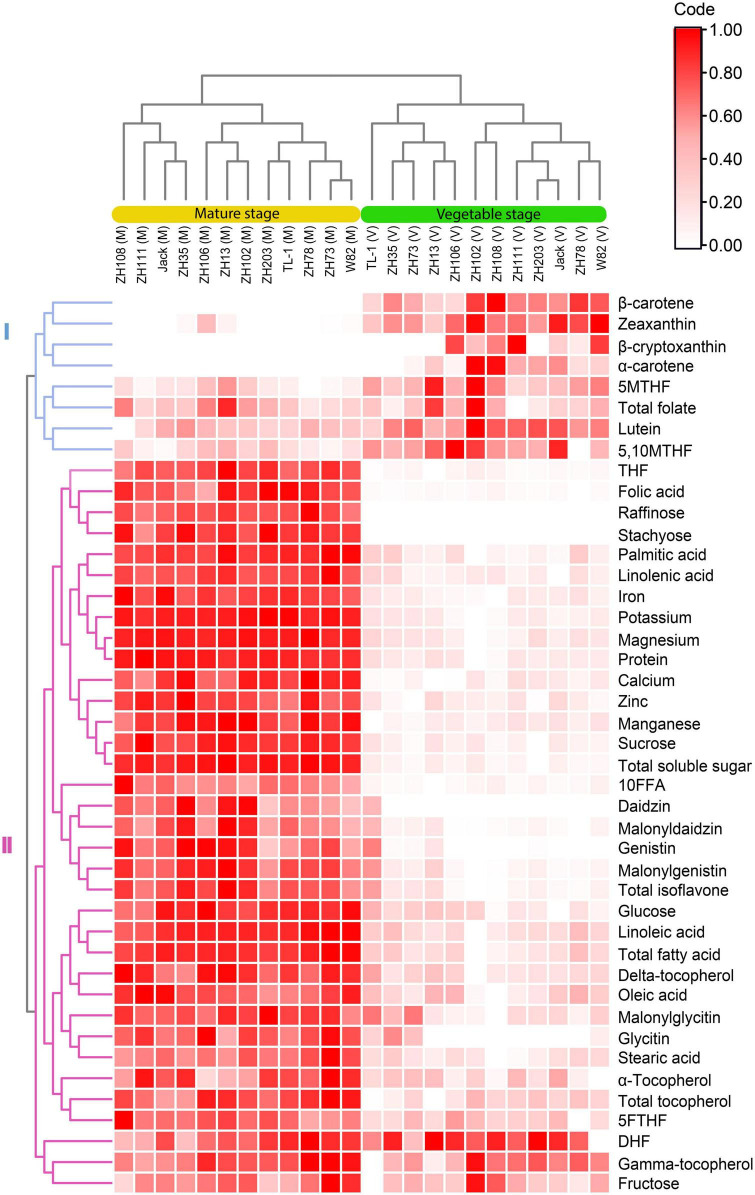
Hierarchical clustering and heatmap of soybean cultivars and quality traits (FW) at the vegetable and mature stages.

In this study, the dendrogram grouped all the cultivars into two clusters based on the vegetable and mature stages (Figure 6). Furthermore, for each stage, the cultivars were clustered into two groups, with each group having sub-groups. The quality traits were also clustered into two, followed by several subgroups. The groupings of all the quality traits were predominantly caused by the differences in their contents at the mature and vegetable stages. In group I, the concentrations of quality traits were mainly higher at the vegetable stage, and in group II, most of the quality traits were higher at the mature stage.

Further, we can identify subgroups based on how distinct the two stages differed from each other. For instance, 5,10MTHF, 5MTHF, total folate, β-carotene, α-carotene, β-cryptoxanthin, lutein, and zeaxanthin clustered into group I but were divided into different subgroups with β-carotene, α-carotene, β-cryptoxanthin, and zeaxanthin falling under one subgroup and the rest being another subgroup. This is because the former appeared to contain significantly lower concentrations at the mature stage. In contrast, the other group, which comprised 5,10MTHF, 5MTHF, lutein, and total folate, was almost the same at both stages despite being significantly different. This indicates that soybean quality traits are discrete and unique at every stage. In group II, the quality traits were further divided into two subgroups. DHF, γ-tocopherol, and fructose belonged to one subgroup as their concentrations at both stages differed narrowly. On the other hand, the other group showed visible contrasting groups at both stages.

**FIGURE 7:**
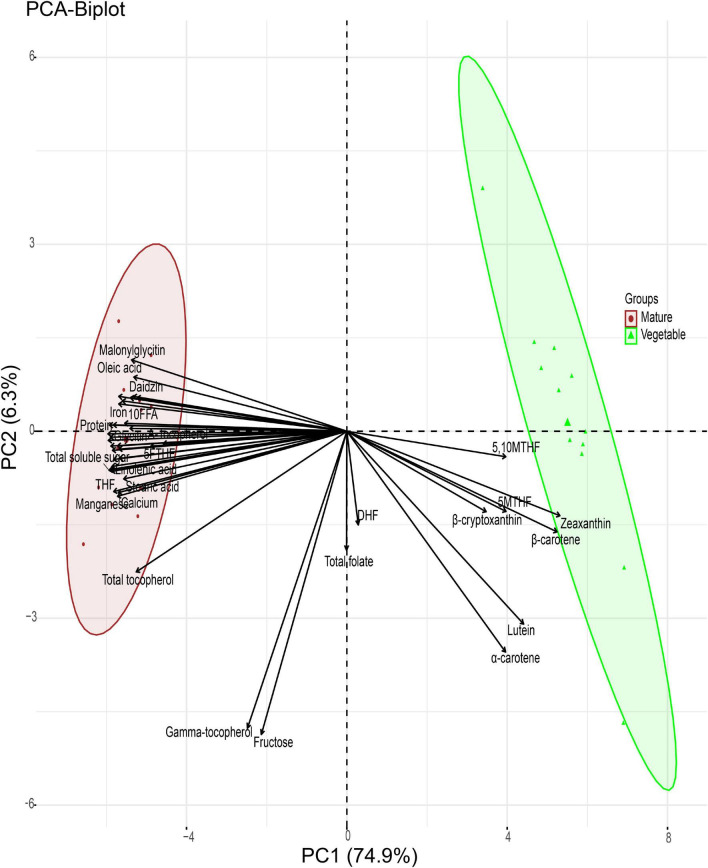
Principal component analysis (PCA) of soybean cultivars and quality traits (FW) at the vegetable and mature stages.

PCA was computed for the quality traits of the 12 soybean seeds at the two different stages. The application of PCA revealed that most soybean quality traits are separated by the maturation stage (Figure 7). The first two principal components (PCs) accounted for 81.20% of the variance observed. PC1 explained 74.90%, whereas PC2 explained 6.30% of the variance. The PCA biplot provided the association of quality traits with the maturation stage. All cultivars were grouped according to their maturation stage, and there was no interaction between cultivars at the vegetable and mature stages. Traits including 5,10MTHF, Zeaxanthin, 5MTHF, β-carotene, β-cryptoxanthin, lutein, and α-carotene were grouped at the vegetable stage consistent with the hierarchical clustering.

Total folate and DHF appeared to be grouped between the two stages. Although total folate and DHF did not group in the hierarchical clustering, the pattern of quality traits at both stages was very similar and could explain why they grouped in the PCA. Similarly, gamma-tocopherol and fructose are grouped between the mature and vegetable stages, which appears to be the same as seen in the hierarchical clustering.

## 4. Discussion

### 4.1. Nutritional content of maodou

In this study, we profiled the nutritional content of maodou. The protein content of maodou ranged from 37.04 to 42.46% (DW) (Figure 2A and Supplementary Table 2). The protein contents observed in this study are consistent with previous studies on vegetable soybean (28, 29). Comparatively, the protein content of maodou in our study is much higher when compared to other legumes such as common bean (16.70–27.20% DW), bambara groundnut (17.00–17.30% DW), cowpea (20.90–24.70% DW), pea (23.30–26.00% DW), and lentils (25.60–28.90% DW) (30, 31), and also staple food and some kinds of meat (32). Furthermore, maodou is available and affordable and thus can be critical to fulfilling the protein requirement of most developing countries.

Total fatty acids ranged from 5.90 to 9.67 g/100 g FW. The unsaturated fatty acids oleic acid, linoleic acid, and linolenic acid contributed a mean of 80.21% of the total fatty acid content, higher than reported for common bean (76%), bambara groundnut (65%), and cowpea (62%) (30). In contrast, the proportion of saturated fatty acids in maodou, palmitic acid, and stearic acid was 19.79%. Linoleic acid and linolenic acid are polyunsaturated fatty acids reported to reduce cholesterol levels in the human blood and the risks of heart diseases. The high composition of polyunsaturated fatty acids indicates that maodou can be an attractive snack for health-conscious people and further protect against disorders.

The average amount of soluble sugar in maodou was 2.47 g/100 g, comprising glucose, fructose, and sucrose. The three free sugars detected at the vegetable stage contribute to the sweetness of maodou, with the highest contributions from sucrose (29, 33). On the other hand, the oligosaccharides, raffinose, and stachyose, which are non-digestible and are linked to abdominal discomfort, were not detected in maodou. Their absence in maodou may contribute to the high digestibility of maodou.

Micronutrients, including carotenoids, folates, and tocopherol, were evaluated in maodou (Supplementary Table 4 and Figures 2D–F). The average total folate content was 462.27 μg/100 g FW, with the average 5MTHF content being 356.18 μg/100 g FW. 5MTHF is the most dominant folate vitamer in maodou, followed by 5FTHF. In maodou, 5MTHF and 5FTHF accounted for 76.40% and 16.70% of total folate content, respectively. Comparatively, the total folate content of maodou was 10–20-fold higher than the folate content in staple crops (rice, maize, and wheat) (34) and other legumes, including pea (17-fold), common bean (2–3-fold), and lentils (3-fold) (35). To the best of our knowledge, this is the first study on the profile of the folate content of maodou. This study confirms that maodou is a rich source of folate, especially the most active vitamer, 5MTHF.

The average total carotenoid content was 3935.41 μg/100 g FW in maodou. The carotenoid contents of the soybean cultivars were consistent with previous studies (36). In maize, rice, and wheat, carotenoid contents have been studied to be 4000 μg/100 g, 6300 μg/100 g, and 200 μg/g, respectively (37), which reveals that the carotenoid contents of maodou in this study are higher than most staple crops, except rice which may contain similar carotenoid amounts as maodou. Furthermore, compared with other legumes, maodou contained relatively higher amounts of total carotenoids than most legumes (14, 38).

The average total tocopherol content of maodou was 181.27 μg/g FW. Although studies on the tocopherol contents of maodou have been limited, the results of our experiment are consistent with a previous study (39). In this study, tocopherol contents of maodou were found to be higher than that of maize (37.53 μg/g) (40), rice (63.29 μg/g) (41), and various legume crops (42).

Total isoflavone content varied from 129.26 to 2359.35 μg/g FW in maodou. Few studies have reported the isoflavone content of maodou (8, 39, 43). Moreover, isoflavone accumulation occurs at the later stages of maturity and explains their low amounts in maodou. Despite their importance, isoflavones contribute to the bitterness and beany taste in soybeans. Thus, their low occurrence in vegetable soybean may prevent the strong beany taste and bitterness and thus increase consumer acceptance of maodou.

Minerals including magnesium, potassium, calcium, manganese, iron, and zinc were profiled in maodou. Our results showed that maodou contains significant amounts of minerals. The mineral contents of maodou in this study are higher than those presented in a previous study on vegetable-type soybean seeds (7). These differences may be due to cultivar differences as vegetable-type soybeans were used in their study and may reveal that the mineral content of vegetable-type soybeans may not be as high as the grain-type soybean. Therefore, new vegetable-type soybean cultivars can be developed by crossing high mineral grain-type cultivars. Further comparison of mineral content with other crops revealed that maodou contained relatively higher amounts of most minerals than other crops. These results are also consistent with the findings presented in the review by Marles (44).

### 4.2. The contribution of maodou to daily nutrition requirements

According to the Food and Agriculture Organization (FAO), sustainable diets have low environmental impacts and contribute to food, nutrition security, and healthy life for present and future generations. Sustainable diets are protective and respectful of biodiversity and ecosystems, culturally acceptable, accessible, economically fair and affordable; nutritionally adequate, safe and healthy; while optimizing natural and human resources (45). In recent years, studies have revealed that plant-based diets help treat various disorders and illnesses. Therefore, sustainable diets comprising higher amounts of plant-based diets, including vegetable soybean, can be a better alternative for the future.

**FIGURE 8:**
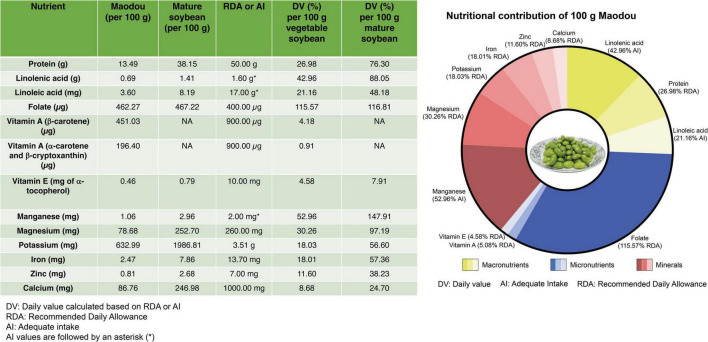
The average nutritional composition of maodou in this study and the corresponding daily values according to the recommended daily allowance (RDA) or adequate intake (AI).

In a typical household, one tablespoonful of maodou may contain about 20–25 seeds which weigh approximately 20 g, and 100 g for one serving is very easy to achieve. Therefore, we evaluate how much nutrients 100 g of fresh maodou can contribute to the recommended daily allowance (RDA) for adults (Figure 8). In instances where no RDAs are set for the nutrients (linoleic acid, linolenic acid, and manganese), we calculated the daily value based on the adequate intake (AI) as recommended by the Institute of Medicine, National academies (46).

#### 4.2.1. Macronutrients

As prescribed by the WHO, the RDA for protein is 50.00 g. In this study, the protein content of maodou ranged between 10.46 and 15.60 g/100 g FW with an average of 13.49 g/100 g (40.28 g/100 g DW), which accounts for 26.98% of the RDA of proteins. This % RDA of protein contributed by maodou is similar to a study in fish (32). On the other hand, meat contains relatively higher amounts of protein, with an average of 22% ranging from 12.30% (duck meat) to 34.50% (chicken breast), contributing to an average of 44% RDA of protein (47).

Fat is a significant source of fuel energy for the body and aids in the absorption of fat-soluble vitamins and other food components such as carotenoids. Although no RDA or AI is set for total fat, AI values have been set for polyunsaturated fatty acids. Polyunsaturated fatty acids, linoleic and linolenic acids are essential fatty acids whose deficiencies are characterized by rough and scaly skin, dermatitis, nerve tissue dysfunctions, and eye problems. The adequate intake range for linoleic acid is 17.00 g/day for active males and 12.00 g/day for females, whereas adequate intake for linolenic acid is 1.60 g/day for males and 1.10 g/day for females (46). In our study, the average linoleic acid content for maodou was 3.60 g/100 g FW, indicating that a 100 g serving of vegetable soybeans can contribute an average of 21.16% of the AI of linoleic acid. Similarly, the linolenic acid content in maodou ranged from 0.58 to 0.81 g/100 g FW, contributing 36.35–50.63% of the AI.

#### 4.2.2. Micronutrients

According to FAO/WHO, the RDA of dietary folates is 400 μg/day (48). In the current study, the average total folate content was 462.27 μg/100 g FW. Therefore, a single serving of 100 g of maodou can supply, on average, 115.57% of RDA. Particularly, the highest content of folate was 685.81 μg/100 g in ZH35, which was 1.7-fold of the RDA of folates. It must also be noted that maodou contained higher proportions (≈77%) of the most active folate vitamer, 5MTHF, and thus folate activity and bioavailability may be increased. Nonetheless, these results are subject to further studies on the bioavailability of maodou folates.

Carotenoids that possess a β-ring have provitamin A activity and can be converted into vitamin A (retinol) by the human body. Beta-carotene with two β-rings has the highest provitamin A activity as one molecule of β-carotene gives two retinol molecules. In contrast, α-carotene or β-cryptoxanthin, with only one β-ring, have half the provitamin A activity compared to β-carotene. Therefore, the 1.00 μg retinol activity requirement (RAE) is equal to 12.00 μg of β-carotene and 24.00 μg of α-carotene and β-cryptoxanthin, with a daily requirement of 900.00 μg of retinol activity equivalents. In this study, the average β-carotene of vegetable soybean was 451.03 μg/100 g FW, corresponding to 37.58 μg RAE. In contrast, the average α-carotene and β-cryptoxanthin content was 196.40 μg/100 g FW, corresponding to 8.18 μg RAE. Cumulatively, vegetable soybean can provide 45.77 μg RAE, which is 5.09% of the daily requirement. Although lutein and zeaxanthin do not have provitamin A activity, they significantly promote eye and skin health and reduce the risk of several chronic diseases. Thus, their presence in maodou will be of critical benefit.

Studies show that α-tocopherol is the only type of vitamin E that human blood can maintain and transfer to cells when needed, and thus the RDA of vitamin E is 10.00 mg of α-tocopherol. In our study, in 100 g of maodou, the average α-tocopherol content was 0.46 mg FW, which shows that the average daily tocopherol value of maodou was 4.58%. α-tocopherol is the lowest occurring tocopherol in maodou and yet the most active. Even in the mature soybean, α-tocopherol maintains to be the least occurring tocopherol. Studies into increasing the α-tocopherol content of soybean may be critical.

#### 4.2.3. Minerals

Iron deficiency is one of the most common micronutrient deficiencies worldwide, which causes anemia, fatigue, and retarded mental growth, and may lead to the risk of casualties during childbirth. The FAO/WHO recommends a daily intake of 13.70 mg of iron (48). The mean iron content in maodou was 2.47 mg/100 g FW, corresponding to 18.01% of the daily value (%DV). Zinc is a trace mineral required for cell proliferation and differentiation and predominantly regulates DNA synthesis and mitosis. The RDA for zinc is 7.00 mg, and as indicated in this study, the zinc content of maodou ranged from 0.69 to 1.18 mg/100 g FW, which means that a serving of 100 g of maodou could provide 9.91 to 16.84% of the daily intake. Higher magnesium intake has been studied to improve T cell activity against infections and cancers (49). The average magnesium content in maodou was 78.68 mg/100 g FW which provides an average of 30.26% DV of magnesium. In a day, the amount of calcium required for adults is 1000 mg. From our study, the calcium content of maodou ranged from 71.89 to 107.57 mg/100 g FW, which can provide 7.19 to 10.76% DV. Potassium is an essential mineral which, in sufficient amounts in the body, will protect against cardiovascular, coronary, and heart diseases. WHO set the RDA for potassium at 3.51 g/day (50). In our study, the average potassium content of maodou was 632.99 mg/100 g, FW, which shows that a 100 g serving of maodou can provide a mean of 18.03% of potassium daily. Due to the rare occurrence of manganese deficiencies, RDAs have not been set. However, the daily AI of manganese ranges from 1.80 to 2.30 mg (average; 2.00 mg), which indicates that maodou can provide an average of 50% AI.

### 4.3. Variations in quality traits at the vegetable and mature stages

We evaluated the content of quality traits at both the vegetable and mature stages to understand the changes in the quality traits at both stages. This will give breeders and consumers an insight of the specific nutritional qualities of the vegetable soybean. As breeders, further studies can be made to exploit the mechanisms of such traits and identify to specific methods to breed such traits. Furthermore, cultivars which are consistently high at both stages can be studied further for breeding programs. In this study, there was no significant difference in the protein content at both stages, which reveals that soybeans are consistently rich in soybean, irrespective of the stage. Cultivars including ZH13 and ZH203, which were consistently higher at both stages could be further used for subsequent studies. The fatty acid content of maodou was higher than the mature soybean. ZH78, which had the highest linoleic acid content in maodou also had linoleic acid contents above average in the mature soybean. Similarly, ZH35, contained the highest contents of linolenic acid at both stages. As such, such cultivars could be selected for further studies. Total soluble sugar content was higher in the mature soybean, but glucose and fructose contents were higher in maodou, whereas no differences were observed for sucrose contents. Sucrose, which is considered as one of the most important traits in maodou was identified to be not affected by stage, which implies that cultivars can be selected, irrespective the stage. Hence, cultivars like ZH13, ZH35, and ZH73 could be further studied.

Total folate and 5MTHF contents were significantly higher in maodou than the mature soybean. 5FTHF, 5,10MTHF, DHF contents were also higher in maodou than in the mature soybean. THF, 10FFA, and FA contents increased in mature soybean. THF is the folate precursor, whereas 10FFA and FA are oxidation products. The changes in the physiology of the soybean seeds may affect folate biosynthesis as well as vitamer distribution in soybean seeds. Further studies to ascertain the reactions that cause changes in the folate vitamer distributions may be necessary. However, ZH73, which was consistently higher in both stages can be selected for further studies. Carotenoids were significantly affected by the stage as most components were hardly detected in mature soybean. Carotenoids may be affected by a plethora of factors leading to their degradation or losses, either through storage or *in planta*. Studies into factors causing the losses of carotenoids during maturity may be important to improve the carotenoid content of mature soybean seeds. Tocopherol contents were significantly higher in maodou, with the exception of δ-tocopherol. Among cultivars, total tocopherol content was the highest in ZH35 at both stages.

Magnesium, potassium, and iron contents differed significantly between maodou and mature soybean. In all these three minerals, higher contents were observed in the mature soybean. On the other hand, manganese, calcium and zinc contents did not differ significantly between the two stages. This implies manganese, calcium, and zinc contents are not affected by stage, whereas magnesium, potassium, and iron could be well studied for high cultivars to improve their contents in maodou.

Isoflavone contents were highly affected by stage. For instance, daidzin, glycitin and genistin detection was relatively low in maodou compared to the mature soybean. Furthermore, total isoflavone was higher in mature soybean. However, a critical factor to consider for maodou is that, despite its beneficial functions, isoflavone may contribute to the bitter taste in soybean and lead to consumer dissatisfaction. Hence, low isoflavone vegetable soybeans may be preferred.

### 4.4. The expression of biosynthesis genes during seeds development

Key enzymes play specific roles in the accumulation of quality traits. In most instances, their expression levels at specific stages may indicate the accumulation of the specific compounds they synthesize. In this study, we studied the expression of key enzymes in the biosynthesis of tocopherol, carotenoid, folate, and isoflavone (Figure 4). Four key genes related to tocopherol were identified, including HPT, HPPT, VTE4, and VTE1. Whereas *GmHPT* and *GmVTE4* decreased with maturity, *GmHPPD* increased with maturity. However, VTE1 had different expression profiles in the two isoforms. Tocopherol phenotypes are higher at the mature stage than in maodou. Gene expression analysis shows relatively different expressions of key tocopherol biosynthesis enzymes. Whereas *GmHPPD* expression may be consistent with the phenotype, *GmHPT*, *GmVTE1.1* and *GmVTE4* somewhat contradicted with the tocopherol contents in the soybean plants at different stages. Thus, functional validation studies may be necessary to confirm the specific effect of these key enzymes at the maodou and mature stages.

For carotenoids, the expression of all isoforms of *GmPSY* decreased with maturity, consistent with the carotenoid contents at the mature and vegetable stages. Being a rate-limiting enzyme of carotenogenesis, high expressions of PSY may have influenced the production of carotenoids in maodou.

All four key folate biosynthesis enzymes, GTPCH1, ADCS, FPGS, and GGH, decreased with maturity in this study. This phenomenon is consistent with studies in tomato and wheat, where GTPCH1, ADCS, and FPGS expressions decreased with ripening or maturity (51–53). In this study, folate contents were higher at the maodou stage, in multiple folds, than at the mature stage. Similarly, the expression of key folate enzymes varied between the maodou and the mature stages. The differential expressions of the key folate enzymes may have contributed to the variation in the phenotype at the two stages.

For isoflavone, *GmIFS* increased in multiple folds at the mature stage, which was consistent with the isoflavone content in soybean cultivars. Thus, the differential expression of IFS at the maodou and mature stages may have contributed to the variation in the isoflavone content of soybean at the two stages.

### 4.5. Correlation analysis of quality traits at vegetable and mature stages

We studied the correlation between quality traits to provide vital information on the interrelationship between the traits. In maodou, there was no correlation between protein and quality traits with the exception of zinc. This observation is consistent with a study by Liu et al. (54), where no correlations were observed between protein and other quality traits in vegetable soybean. On the other hand, at the mature stage, negative correlations were seen between protein and other quality traits (δ-tocopherol, total tocopherol, DHF, linolenic acid, linoleic acid, and total fatty acid). The negative correlation between protein, linoleic acid, and linolenic acid is consistent with a previous study (55). Similarly, the negative correlation between protein and total tocopherol has been reported (15). It is also well established that proteins are negatively correlated with oil, which is positively correlated with total fatty acids. Hence, the negative correlation is expected. Folates were highly correlated with soluble sugar in maodou but correlation was reduced in the mature stage. One-carbon metabolism which is supported by folate metabolism derives units from serine and glycine. Serine can be synthesized *de novo* from glucose which is formed from sucrose (56). This complex cycle may lead to a disruption in the homeostasis of folates and soluble sugars, especially at different maturity stages, leading to changes in associations. Folates and isoflavones have close biosynthetic pathways. Thus, changes in fluxes at different stages may lead to different associations. Isoflavone accumulation occurs with maturity and in this study, whereas no correlations were found between isoflavone and folate in maodou, positive correlations were observed in the mature soybean seed. This may have been caused by differences in accumulations across stages. Such correlations may be carefully studied for future breeding purposes.

## 5. Conclusion

Malnutrition is a major issue, and identifying sustainable sources of nutrition may be critical for the future. In this study, we profiled the nutritional content of maodou. In general, from this study, we identified that maodou is a rich source of proteins, folate, polyunsaturated fatty acids, minerals, carotenoids, and tocopherols. By evaluating the daily value of maodou, we identified that a 100 g serving of maodou could provide 26.98% of the RDA of proteins, and 21.16 and 42.96% of the AI of essential fatty acids, linoleic acid, and linolenic acid, respectively. Additionally, a serving of 100 g of maodou can contribute to 115.57% DV of folates, 5.08% DV of vitamin A and 4.58% DV of vitamin E. Regarding minerals, we found that a 100 g serving of maodou can contribute to an average of 30.26% DV of magnesium, 18.03% DV of potassium, 18.03% DV of iron, 11.60% DV of zinc and 8.68% DV of calcium. Furthermore, our studies revealed that maodou could contribute to 52.96% of the AI of manganese.

RNA-seq studies also showed differential expression of key biosynthesis enzymes for tocopherol, carotenoid, folate, and isoflavone, which may have influenced their differences at the maodou and mature stages. By analyzing the correlation between quality traits at both maodou and mature stages, we identified a higher number of significant associations in maodou than the mature soybean. Furthermore, we identified associations consistent across stages and associations exclusive to either maodou or mature soybean. The information will benefit consumers’ food choices, assist in breeding, and enhance further research into soybean nutritional quality. Furthermore, this study highlights that soybean contains many nutrients yet to be exploited with mechanisms understood at the vegetable stage. Altogether, this study reveals that soybean is a storehouse of nutrients and can be utilized to fight malnutrition.

## Data availability statement

The datasets presented in this study can be found in online repositories. The data presented in this study are deposited in the Genome Sequence Archive repository, accession number GSA: CRA008483.

## Author contributions

KGA-B and SRZ: formal analysis, investigation, methodology, software, writing—original draft, review and editing, and data curation. SBZ, ANK, AS, AMA, JQ, MA, CM, YF, HF, YL, and JL: investigation and methodology. BL and JS: conceptualization, funding acquisition, project administration, supervision, resources, and writing—review and editing. All authors contributed to the article and approved the submitted version.

## Footnotes

1
https://phytozome-next.jgi.doe.gov

2
https://cran.r-project.org/web/packages/agricolae

3
https://cran.r-project.org/web/packages/FactoMineR/index.html
