# Supplementary material for: The nutritional composition of the vegetable soybean (maodou) and its potential in combatting malnutrition
Source: Front Nutr. 2023 Jan 5;9:1034115. doi: 10.3389/fnut.2022.1034115 (PMC9849953; doi:10.3389/fnut.2022.1034115)
Supplement: Supplementary file 1 [file Table_1.DOCX]

**Supplementary Table S1**. Characteristics of cultivars used in this study

| **Cultivar** | **Maturity Group** | **Origin** | **Agronomic and Quality Characteristics** |
| --- | --- | --- | --- |
| ZH13 | MGIII | China | Planting area over 6.67 million hectares |
| ZH35 | MGIII | China | High yield, record yield 6320.55 kg/hm^2^ in 2012 |
| ZH73 | MGIII | China | High yield |
| ZH78 | MGIII | China | Low off-flavour (*lox-3*) |
| ZH102 | MGIII | China | Low off-flavour (*lox-2)* |
| ZH106 | MGIII | China | High oil |
| ZH108 | MGIII | China | High yield |
| ZH111 | MGIII | China | High yield |
| ZH203 | MGIII | China | Low off-flavor (*lox-2)* |
| TL-1 | MGII | China | Resistant to soybean mosaic virus |
| Jack | MGII | USA | Resistant to cyst nematode race 3 and 4 |
| W 82 | MGIII | USA | Phytophthora rot resistance |

**Supplementary Table S2**. Macronutrient composition of maodou on fresh weight (FW) basis

| **Cultivar** | **Protein (g/100g)** | **Palmitic acid (g/100g)** | **Stearic acid （g/100g）** | **Oleic acid (g/100g)** | **Linoleic acid (g/100g)** | **Linolenic acid (g/100g)** | **Total fatty acid (g/100g)** | **Fructose (g/100g)** | **Glucose (g/100g)** | **Sucrose (g/100g)** | **Total soluble sugar (g/100g)** |
| --- | --- | --- | --- | --- | --- | --- | --- | --- | --- | --- | --- |
| **ZH 13** | 15.25±0.31 | 1.02±0.04 | 0.44±0.02 | 2.50±0.17 | 3.27±0.18 | 0.64±0.04 | 7.87±0.44 | 0.20±0.02 | 0.24±0.02 | 2.33±0.14 | 2.78±0.04 |
| **ZH 35** | 10.46±0.27 | 0.93±0.05 | 0.46±0.02 | 1.47±0.11 | 2.35±0.18 | 0.70±0.06 | 5.90±0.43 | 0.36±0.02 | 0.23±0.02 | 2.24±0.10 | 2.83±0.08 |
| **ZH 73** | 11.29±0.07 | 1.00±0.06 | 0.39±0.02 | 1.35±0.13 | 2.99±0.25 | 0.72±0.06 | 6.46±0.51 | 0.30±0.00 | 0.16±0.01 | 1.85±0.06 | 2.31±0.03 |
| **ZH 78** | 13.44±1.25 | 1.25±0.05 | 0.50±0.07 | 2.56±0.11 | 4.62±0.24 | 0.75±0.04 | 9.67±0.48 | 0.16±0.00 | 0.20±0.02 | 1.96±0.08 | 2.32±0.00 |
| **ZH 102** | 13.28±0.13 | 1.02±0.04 | 0.44±0.01 | 1.72±0.06 | 3.59±0.18 | 0.69±0.03 | 7.46±0.32 | 0.20±0.01 | 0.19±0.01 | 1.70±0.01 | 2.08±0.02 |
| **ZH 106** | 14.11±0.14 | 1.16±0.10 | 0.48±0.04 | 2.49±0.21 | 3.66±0.31 | 0.67±0.06 | 8.46±0.72 | 0.20±0.00 | 0.23±0.01 | 2.01±0.05 | 2.43±0.01 |
| **ZH 108** | 13.96±0.22 | 1.24±0.07 | 0.53±0.02 | 1.95±0.13 | 4.51±0.30 | 0.77±0.05 | 9.00±0.57 | 0.21±0.01 | 0.21±0.00 | 2.05±0.04 | 2.47±0.03 |
| **ZH 111** | 13.22±0.04 | 1.04±0.11 | 0.46±0.06 | 1.68±0.15 | 3.52±0.33 | 0.64±0.06 | 7.34±0.71 | 0.23±0.01 | 0.23±0.03 | 1.78±0.07 | 2.24±0.05 |
| **ZH 203** | 14.22±0.01 | 0.98±0.11 | 0.40±0.03 | 1.60±0.15 | 3.43±0.33 | 0.64±0.07 | 7.05±0.69 | 0.24±0.04 | 0.19±0.01 | 2.01±0.23 | 2.45±0.04 |
| **Jack** | 13.71±0.31 | 0.97±0.06 | 0.47±0.04 | 2.15±0.13 | 3.49±0.19 | 0.58±0.03 | 7.66±0.46 | 0.19±0.00 | 0.16±0.02 | 2.20±0.03 | 2.55±0.06 |
| **TL-1** | 15.60±0.06 | 1.23±0.02 | 0.48±0.01 | 2.31±0.07 | 4.04±0.17 | 0.81±0.04 | 8.87±0.29 | 0.13±0.01 | 0.28±0.00 | 2.37±0.13 | 2.78±0.07 |
| **W 82** | 13.36±0.13 | 1.03±0.04 | 0.49±0.02 | 2.08±0.08 | 3.71±0.13 | 0.66±0.02 | 7.96±0.30 | 0.24±0.01 | 0.17±0.01 | 1.94±0.12 | 2.35±0.01 |
| **Average** | 13.49 | 1.07 | 0.46 | 1.99 | 3.60 | 0.69 | 7.81 | 0.22 | 0.21 | 2.04 | 2.47 |

**Supplementary Table S3**. Macronutrient composition of mature soybean on fresh weight (FW) basis

| **Cultivar** | **Protein (g/100g)** | **Palmitic acid (g/100g)** | **Stearic acid （g/100g）** | **Oleic acid (g/100g)** | **Linoleic acid (g/100g)** | **Linolenic acid (g/100g)** | **Total fatty acid (g/100g)** | **Fructose (g/100g)** | **Glucose (g/100g)** | **Raffinose (g/100g)** | **Stachyose (g/100g)** | **Sucrose (g/100g)** | **Total soluble sugar (g/100g)** |
| --- | --- | --- | --- | --- | --- | --- | --- | --- | --- | --- | --- | --- | --- |
| **ZH 13** | 42.07±0.14 | 1.89±0.02 | 0.68±0.00 | 4.40±0.06 | 6.97±0.09 | 1.29±0.02 | 15.22±0.20 | 0.26±0.01 | 0.34±0.03 | 0.34±0.03 | 1.24±0.23 | 6.41±0.06 | 8.6±0.01 |
| **ZH 35** | 36.10±0.35 | 2.24±0.05 | 0.92±0.02 | 3.67±0.08 | 9.21±0.23 | 1.69±0.04 | 17.73±0.43 | 0.38±0.03 | 0.41±0.03 | 0.44±0.08 | 1.93±0.22 | 5.67±0.06 | 8.83±0.04 |
| **ZH 73** | 36.26±0.73 | 2.19±0.02 | 0.65±0.01 | 3.38±0.04 | 8.34±0.07 | 1.51±0.02 | 16.08±0.16 | 0.30±0.01 | 0.40±0.01 | 0.47±0.02 | 2.09±0.28 | 6.11±0.09 | 9.38±0.04 |
| **ZH 78** | 38.66±0.24 | 1.89±0.03 | 0.71±0.02 | 3.59±0.04 | 8.31±0.10 | 1.45±0.02 | 15.94±0.21 | 0.28±0.02 | 0.47±0.05 | 0.41±0.07 | 1.81±0.18 | 5.83±0.03 | 8.80±0.13 |
| **ZH 102** | 39.65±0.26 | 2.00±0.01 | 0.72±0.01 | 4.29±0.03 | 7.97±0.07 | 1.36±0.02 | 16.34±0.12 | 0.27±0.01 | 0.45±0.01 | 0.39±0.03 | 1.87±0.30 | 5.02±0.07 | 8.00±0.02 |
| **ZH 106** | 36.57±0.26 | 2.21±0.05 | 0.77±0.02 | 4.03±0.10 | 9.07±0.20 | 1.33±0.03 | 17.41±0.40 | 0.34±0.02 | 0.46±0.01 | 0.34±0.02 | 1.91±0.29 | 5.35±0.04 | 8.40±0.02 |
| **ZH 108** | 38.39±0.56 | 2.03±0.04 | 0.70±0.05 | 2.87±0.03 | 7.98±0.07 | 1.38±0.01 | 14.95±0.20 | 0.20±0.00 | 0.42±0.02 | 0.42±0.04 | 2.46±0.39 | 5.41±0.12 | 8.90±0.03 |
| **ZH 111** | 37.78±0.19 | 2.07±0.06 | 0.70±0.03 | 3.00±0.11 | 8.11±0.25 | 1.38±0.04 | 15.26±0.49 | 0.23±0.05 | 0.42±0.00 | 0.42±0.08 | 1.93±0.47 | 5.42±0.05 | 8.42±0.05 |
| **ZH 203** | 39.17±0.36 | 1.94±0.00 | 0.66±0.01 | 3.49±0.02 | 8.36±0.04 | 1.33±0.01 | 15.77±0.05 | 0.25±0.01 | 0.42±0.02 | 0.44±0.01 | 2.36±0.26 | 5.14±0.07 | 8.60±0.02 |
| **Jack** | 36.53±0.19 | 2.01±0.03 | 0.77±0.02 | 3.18±0.05 | 8.83±0.13 | 1.45±0.02 | 16.22±0.24 | 0.28±0.01 | 0.42±0.03 | 0.60±0.23 | 2.13±0.22 | 5.83±0.04 | 9.26±0.03 |
| **TL-1** | 38.71±0.34 | 1.88±0.01 | 0.65±0.01 | 3.83±0.02 | 6.85±0.01 | 1.41±0.00 | 14.62±0.05 | 0.18±0.01 | 0.35±0.00 | 0.43±0.03 | 2.29±0.15 | 4.87±0.05 | 8.11±0.04 |
| **W 82** | 37.95±0.22 | 1.94±0.02 | 0.76±0.01 | 3.27±0.05 | 8.28±0.12 | 1.34±0.02 | 15.60±0.22 | 0.30±0.01 | 0.38±0.01 | 0.41±0.08 | 1.68±0.65 | 5.67±0.06 | 8.44±0.08 |
| **Average** | 38.15 | 2.02 | 0.72 | 3.58 | 8.19 | 1.41 | 15.93 | 0.27 | 0.41 | 0.43 | 1.97 | 5.56 | 8.65 |

**Supplementary Table S4**. Micronutrient composition of Maodou on fresh weight (FW) basis

| **Cultivar** | **THF(µg/ 100g)** | **5MTHF(µg/ 100g)** | **5,10MTHF(µg/ 100g)** | **10FFA (µg/ 100g)** | **5FTHF (µg/ 100g)** | **DHF (µg/ 100g)** | **FA (µg/ 100g)** | **Total folate (µg/ 100g)** | **β-carotene (µg/100g)** | **Lutein (µg/100g)** | **Zeaxanthin (µg/100g)** | **β-cryptoxanthin (µg/100g)** | **α-carotene (µg/ 100g)** | **Total carotenoid (µg/ 100g)** | **Delta tocopherol (µg/100g)** | **Gamma tocopherol (µg/100g)** | **Alpha tocopherol (µg/g100)** | **Total tocopherol**  **(µg/100g)** |
| --- | --- | --- | --- | --- | --- | --- | --- | --- | --- | --- | --- | --- | --- | --- | --- | --- | --- | --- |
| **ZH 13** | 10.76±1.43 | 514.18±20.45 | 11.24±0.09 | 3.27±0.26 | 64.88±3.64 | 3.60±0.72 | 1.32±0.41 | 609.25±17.66 | 173.91±13.6 | 2197.02±73.72 | 63.54±2.50 | ND | 48.47±4.35 | 2482.94±952.70 | 4734.84±217.71 | 10216.62±269.43 | 543.26±27.65 | 15494.73±486.97 |
| **ZH 35** | 15.34±2.46 | 558.71±20.55 | 12.34±0.43 | 4.59±0.84 | 90.62±3.61 | 2.94±0.78 | 1.26±0.33 | 685.81±25.16 | 652.27±31.21 | 5259.64±8.32 | 241.68±1.62 | 72.98±6.24 | 183.77±8.46 | 6410.35±2234.29 | 2050.4±44.2 | 18346.39±336.63 | 478.11±103.73 | 20874.9±315.52 |
| **ZH 73** | 13.67±0.62 | 381.50±1.90 | 9.83±1.23 | 5.62±1.27 | 70.18±6.79 | 3.34±1.30 | 1.82±1.21 | 485.96±10.93 | 847.87±17.84 | 3645.96±12.63 | 144.99±4.37 | 127.46±2.86 | 175.39±3.31 | 4941.66±1516.24 | 3050.67±102.11 | 14940.76±52.51 | 334.72±177.13 | 18326.15±189.5 |
| **ZH 78** | 11.76±1.49 | 351.74±1.71 | 5.66±0.20 | 4.17±0.79 | 40.59±2.34 | 2.90±1.34 | 0.32±0.04 | 417.14±3.38 | 674.92±13.48 | 2701.66±46.68 | 181.25±1.64 | 22.56±0.63 | 28.24±3.55 | 3608.63±1138.44 | 3614.66±128.46 | 15666.41±453.04 | 366±202.97 | 19647.07±572.62 |
| **ZH 102** | 11.88±2.24 | 274.92±14.26 | 9.13±1.06 | 4.68±0.95 | 73.72±1.69 | 3.58±0.45 | 0.74±0.42 | 378.66±15.04 | 472.06±5.84 | 3819.48±46.95 | 118.61±0.17 | ND | 80.99±5.27 | 4491.15±1643.00 | 3349.03±205.04 | 15883.25±170.12 | 416.58±134.97 | 19648.86±270.75 |
| **ZH 106** | 13.07±1.04 | 329.95±9.65 | 14.27±1.3 | 4.01±0.41 | 108.16±8.78 | 3.30±0.53 | 0.93±0.52 | 473.68±5.48 | 150.00±7.44 | 2630.81±4.04 | 156.12±1.01 | 169.25±4.75 | 9.50±3.56 | 3115.68±1124.20 | 3980.06±161.91 | 12895.01±565.52 | 361.22±16.98 | 17236.29±741.48 |
| **ZH 108** | 12.87±1.93 | 272.32±8.92 | 8.93±2.25 | 3.89±1.48 | 65.41±5.94 | 3.34±1.97 | 0.66±0.71 | 367.42±9.45 | 448.07±4.73 | 2977.07±37.42 | 127.53±2.13 | ND | ND | 3552.68±1280.20 | 3227.34±310.05 | 12781.68±389.55 | 519.26±74.68 | 16528.29±728.14 |
| **ZH 111** | 13.01±1.64 | 319.52±5.36 | 9.57±1.69 | 4.01±1.04 | 93.06±4.87 | 2.25±0.62 | 0.94±0.33 | 442.37±5.89 | 358.91±19.34 | 3503.54±4.58 | 121.76±5.83 | ND | 7.59±2.05 | 3991.80±1519.75 | 3984.03±217.24 | 13933.16±847.25 | 538.9±33.44 | 18456.08±1097.32 |
| **ZH 203** | 11.67±1.75 | 241.47±8.23 | 9.44±0.41 | 3.89±1.29 | 73.71±1.77 | 2.93±0.74 | 0.96±0.06 | 344.06±9.91 | 463.03±11.02 | 3448.53±18.39 | 152.33±6.5 | 229.83±4.87 | 76.39±3.26 | 4370.10±1446.46 | 3171.13±114.11 | 15127.19±506.07 | 563.72±150.14 | 18862.04±706.35 |
| **Jack** | 12.53±0.76 | 293.11±6.00 | 13.03±0.37 | 3.94±1.14 | 91.32±0.62 | 3.29±2.73 | 1.07±0.22 | 418.28±9.21 | 425.81±24.11 | 3733.51±19.81 | 224.33±4.78 | 52.4±5.90 | 94.38±5.38 | 4530.43±1587.25 | 3248.84±65.09 | 14524.91±315.30 | 624.68±65.12 | 18398.43±313.03 |
| **TL-1** | 11.20±1.07 | 349.05±3.66 | 9.93±1.21 | 4.59±1.83 | 64.4±1.32 | 2.64±0.94 | 0.88±0.16 | 442.68±1.75 | 169.58±7.42 | 1376.91±19.59 | 72.44±6.03 | ND | ND | 1618.93±592.81 | 5966±114.00 | 9418.96±39.17 | 441.62±24.53 | 15509.57±594.87 |
| **W 82** | 12.81±2.17 | 387.65±9.3 | 8.72±1.92 | 5.27±2.02 | 64.81±0.91 | 1.61±0.41 | 1.1±0.18 | 481.96±7.67 | 575.91±6.33 | 3064.76±17.49 | 250.73±2.21 | 178.71±7.42 | 40.41±0.3 | 4110.52±1269.01 | 3745.44±78.98 | 14493.42±509.69 | 307.75±147.33 | 18546.6±484.6 |
| **Average** | 12.55 | 356.18 | 10.17 | 4.33 | 75.07 | 2.98 | 1.00 | 462.27 | 451.03 | 3196.57 | 154.61 | 121.89 | 74.51 | 3935.41 | 3676.87 | 14018.98 | 457.98 | 18127.42 |

ND- not detected

**Supplementary Table S5.** Micronutrient composition of mature soybean on fresh weight (FW) basis

| **Cultivar** | **THF(µg/ 100g)** | **5MTHF(µg/ 100g)** | **5,10MTHF(µg/ 100g)** | **10FFA**  **(µg/ 100g)** | **5FTHF**  **(µg/ 100g)** | **DHF (µg/ 100g)** | **FA (µg/ 100g)** | **Total folate (µg/ 100g)** | **Lutein (µg/100g)** | **Zeaxanthin (µg/100g)** | **Total carotenoids** | **Delta tocopherol (µg/100g)** | **Gamma tocopherol (µg/100g)** | **Alpha tocopherol (µg/g100)** | **Total tocopherol (µg/100g)** |
| --- | --- | --- | --- | --- | --- | --- | --- | --- | --- | --- | --- | --- | --- | --- | --- |
| **ZH 13** | 50.44±1.66 | 185.94±1.60 | 6.17±1.00 | 21.01±2.38 | 122.79±8.81 | 2.44±0.85 | 20.75±1.29 | 409.54±8.43 | 1178.28±5.08 | ND | 1178.28±5.08 | 9535.21±138.00 | 13580.29±207.07 | 982.58±27.83 | 24098.08±351.93 |
| **ZH 35** | 56.14±4.98 | 184.23±4.10 | 6.04±0.67 | 18.96±3.16 | 110.38±1.00 | 3.27±1.70 | 22.23±0.51 | 401.25±8.22 | 1833.2±30.71 | 1.74±0.83 | 1834.94±1295.04 | 9879.92±67.63 | 18817.7±181.75 | 1041.52±46.74 | 29739.14±277.44 |
| **ZH 73** | 65.18±2.58 | 359.75±3.46 | 9.15±2.22 | 20.41±2.20 | 149.24±5.59 | 2.98±0.93 | 28.14±1.86 | 634.84±6.50 | 1717.77±22.41 | 13.72±2.43 | 1731.49±1204.95 | 10632.37±209.54 | 16241.45±87.12 | 583.29±25.58 | 27457.11±262.23 |
| **ZH 78** | 51.28±12.42 | 293.96±6.07 | 8.47±1.33 | 19.13±2.24 | 141.07±9.08 | 2.82±1.33 | 12.92±1.08 | 529.64±6.09 | 2101.01±43.64 | 83.32±0.38 | 2184.33±1246.72 | 10306.39±125.98 | 17433.22±293.02 | 443.06±14.74 | 28182.67±403.38 |
| **ZH 102** | 46.70±3.58 | 219.33±2.58 | 5.83±0.42 | 23.56±1.05 | 128.70±4.26 | 3.05±0.55 | 21.06±1.72 | 448.23±6.85 | 2359.6±36 | ND | 2359.60±1668.49 | 7075.58±276.32 | 14157.26±151.08 | 807.06±47.00 | 22039.9±418.14 |
| **ZH 106** | 48.44±5.45 | 201.9±1.76 | 6.91±0.50 | 16.59±0.61 | 120.26±3.57 | 3.20±1.40 | 21.24±0.43 | 418.54±2.55 | 1660.83±16.24 | 3.89±0.14 | 1664.72±1171.64 | 9337.04±75.99 | 18160.07±123.53 | 918.92±53.09 | 28416.03±200.23 |
| **ZH 108** | 55.96±1.10 | 210.27±0.74 | 8.64±0.87 | 22.67±1.72 | 142.09±0.11 | 3.17±1.80 | 30.42±5.72 | 473.23±7.00 | 1337.52±14.86 | ND | 1337.52±945.77 | 7504.78±108.02 | 15771.39±157.51 | 886.2±16.93 | 24162.37±232.47 |
| **ZH 111** | 44.90±2.33 | 199.54±8.12 | 6.99±0.23 | 22.46±2.03 | 131.23±1.25 | 3.30±0.39 | 29.81±2.27 | 438.24±8.75 | 1325.72±26.63 | ND | 1325.72±937.43 | 9050.69±93.90 | 16064.56±143.25 | 843.11±28.84 | 25958.36±262.17 |
| **ZH 203** | 47.00±1.16 | 218.13±2.29 | 7.35±0.24 | 19.26±1.32 | 124.24±1.84 | 2.25±1.09 | 17.22±1.78 | 435.45±2.12 | 2719.15±32.59 | 8.13±1.02 | 2727.28±1916.98 | 6628.79±56.68 | 14689.01±273.22 | 927.37±18.92 | 22245.17±321.17 |
| **Jack** | 49.51±1.44 | 171.53±2.14 | 6.44±0.28 | 20.41±1.35 | 102.18±4.20 | 3.6±0.40 | 27.14±3.65 | 380.81±7.21 | 2291.18±17.17 | ND | 2291.18±1620.11 | 7497.36±41.70 | 18877.38±183.34 | 777.11±35.41 | 27151.85±260.39 |
| **TL-1** | 41.46±2.42 | 237.57±0.68 | 7.89±0.62 | 34.69±1.08 | 184.39±5.75 | 2.30±0.50 | 25.73±2.98 | 534.04±6.51 | 301.99±4.48 | ND | 301.99±213.54 | 10881.69±302.13 | 14566.72±70.36 | 643.58±28.10 | 26091.98±333.47 |
| **W 82** | 51.09±4.27 | 271.08±2.69 | 7.44±0.74 | 17.03±1.27 | 129.23±6.92 | 2.84±0.08 | 24.18±1.29 | 502.89±7.75 | 1669.57±47.09 | ND | 1669.57±1180.56 | 9307.03±494.51 | 15855.99±376.97 | 636.89±39.18 | 25799.91±809.74 |
| **Average** | 50.67 | 229.44 | 7.28 | 21.35 | 132.15 | 2.93 | 23.40 | 467.22 | 1707.98 | 22.16 | 1717.22 | 8969.74 | 16184.59 | 790.89 | 25945.21 |

ND- Not detected

**Supplementary Table S6.** Isoflavone content of Maodou on fresh weight (FW) basis

| **Cultivar** | **Daidzin (µg/g)** | **Glycitin (µg/g)** | **Genistin (µg/g)** | **Malonyldaidzin (µg/g)** | **Malonylglycitin (µg/g)** | **Malonylgenistin (µg/g)** | **Total isoflavone (µg/g)** |
| --- | --- | --- | --- | --- | --- | --- | --- |
| **ZH 13** | ND | ND | 21.95±1.22 | 232.72±7.83 | 111.98±23.91 | 565.15±13.03 | 931.8±38.71.00 |
| **ZH 35** | ND | ND | ND | 35.29±1.12 | 73.16±0.24 | 20.81±0.06 | 129.26±0.82 |
| **ZH 73** | ND | ND | ND | 60.87±7.96 | 46.69±3.39 | 39.69±6.21 | 147.24±9.85 |
| **ZH 78** | ND | ND | ND | 33.74±4.30 | 80.72±7.64 | 95.51±4.03 | 209.97±10.51 |
| **ZH 102** | ND | ND | 1.79±0.40 | 122.67±6.97 | 141.01±3.94 | 189.59±0.69 | 455.06±8.04 |
| **ZH 106** | ND | ND | ND | 40.11±9.47 | 80.76±15.35 | 109.9±4.31 | 230.77±27.25 |
| **ZH 108** | ND | 25.59±0.54 | 3.89±1.42 | 130.71±5.90 | 239.09±28.36 | 251.11±12.68 | 650.40±47.92 |
| **ZH 111** | ND | 15.36±1.93 | 5.78±1.01 | 144.93±6.19 | 365.76±2.50 | 199.54±2.29 | 731.37±7.46 |
| **ZH 203** | ND | ND | ND | 78.24±4.41 | 143.95±5.03 | 118.05±5.96 | 340.24±15.31 |
| **Jack** | ND | ND | ND | 66.25±6.93 | 155.64±11.64 | 66.4±4.68 | 288.29±21.29 |
| **TL-1** | 40.05±3.24 | 10.36±2.66 | 94.31±3.38 | 649.71±22.55 | 367.34±2.26 | 1197.58±48.63 | 2359.35±73.21 |
| **W 82** | ND | 4.05±2.72 | ND | 128.32±4.05 | 172.6±4.47 | 129.63±1.84 | 434.59±13.08 |
| **Average** | 40.05 | 13.84 | 25.55 | 143.63 | 164.89 | 248.58 | 575.69 |

ND- not detected

**Supplementary Table S7**. Isoflavone content of mature soybean on fresh weight basis

| **Cultivar** | **Daidzin (µg/g)** | **Glycitin (µg/g)** | **Genistin (µg/g)** | **Malonyldaidzin (µg/g)** | **Malonylglycitin (µg/g)** | **Malonylgenistin (µg/g)** | **Total isoflavone (µg/g)** |
| --- | --- | --- | --- | --- | --- | --- | --- |
| **ZH 13** | 61.00±5.39 | 39.96±5.44 | 98.13±0.32 | 798.29±10.43 | 396.85±9.35 | 1484.58±14.06 | 2878.81±13.82 |
| **ZH 35** | 49.72±2.2 | 47.34±2.35 | 127.19±4.13 | 880.90±2.31 | 503.58±23.77 | 1883.46±16.8 | 3492.18±12.60 |
| **ZH 73** | 100.96±3.65 | 20.86±0.30 | 158.99±3.30 | 1719.94±5.70 | 510.40±53.03 | 2450.64±5.40 | 4961.79±60.30 |
| **ZH 78** | 57.07±2.04 | 49.35±3.91 | 167.41±4.29 | 836.26±16.39 | 368.16±14.00 | 2174.49±1.65 | 3652.74±23.91 |
| **ZH 102** | 72.27±2.23 | 28.26±2.52 | 112.93±0.94 | 1242.48±7.06 | 405.42±5.18 | 1581.73±6.37 | 3443.08±9.91 |
| **ZH 106** | 33.57±2.10 | 34.75±4.07 | 72.97±4.14 | 662.48±7.41 | 337.09±28.94 | 1354.38±25.95 | 2495.24±56.07 |
| **ZH 108** | 30.56±1.29 | 33.24±0.98 | 45.67±2.24 | 769.21±11.09 | 597.20±28.20 | 1182.73±1.83 | 2658.6±44.43 |
| **ZH 111** | 56.57±2.01 | 26.50±1.90 | 81.06±3.08 | 1112.21±5.41 | 461.65±0.88 | 1788.50±25.17 | 3526.4832.24 |
| **ZH 203** | 110.37±2.65 | 31.15±3.15 | 170.24±2.15 | 1561.62±1.51 | 445.64±19.19 | 2074.57±2.84 | 4393.59±21.01 |
| **Jack** | 55.53±0.37 | 35.53±5.28 | 107.41±0.65 | 915.37±7.49 | 477.62±15.14 | 1786.42±3.05 | 3377.88±27.92 |
| **TL-1** | 76.26±4.14 | 31.90±1.46 | 158.34±6.04 | 1123.16±8.34 | 495.12±9.76 | 2035.14±19.38 | 3919.92±21.65 |
| **W 82** | 108.12±2.03 | 37.81±11.66 | 141.57±1.59 | 1465.07±11.04 | 462.61±28.8 | 1989.77±4.21 | 4204.96±41.86 |
| **Average** | 67.67 | 34.72 | 120.16 | 1090.58 | 455.11 | 1815.53 | 3583.77 |

**Supplementary Table S8.** Mineral content of Maodou on fresh weight (FW) basis

| **Cultivar** | **Magnesium (Mg) (mg/100 g)** | **Potassium (K)**  **(mg/100 g)** | **Calcium (Ca)**  **(mg/100 g)** | **Manganese (Mn) (mg/100 g)** | **Iron (Fe) (mg/100 g)** | **Zinc (Zn) (mg/100 g)** |
| --- | --- | --- | --- | --- | --- | --- |
| **ZH 13** | 84.42±0.01 | 683.28±0.07 | 71.89±0.01 | 1.08±0.11 | 2.20±0.21 | 1.18±0.06 |
| **ZH 35** | 55.23±0.03 | 501.36±0.28 | 97.98±0.05 | 0.98±0.55 | 2.05±1.69 | 0.92±0.36 |
| **ZH 73** | 61.44±0.02 | 532.48±0.11 | 77.80±0.01 | 1.08±0.34 | 1.86±0.66 | 0.86±0.11 |
| **ZH 78** | 87.99±0.01 | 611.73±0.03 | 82.35±0.00 | 1.03±0.07 | 2.98±0.13 | 0.95±0.00 |
| **ZH 102** | 92.52±0.00 | 685.38±0.01 | 100.33±0.00 | 1.11±0.04 | 2.61±0.09 | 0.71±0.01 |
| **ZH 106** | 71.85±0.01 | 548.89±0.06 | 72.15±0.01 | 1.10±0.20 | 2.16±0.77 | 0.95±0.02 |
| **ZH 108** | 86.02±0.00 | 703.02±0.02 | 75.14±0.00 | 0.97±0.01 | 2.55±0.15 | 0.79±0.04 |
| **ZH 111** | 85.04±0.01 | 695.45±0.02 | 85.16±0.01 | 0.91±0.01 | 2.62±0.20 | 0.69±0.08 |
| **ZH 203** | 68.51±0.01 | 656.01±0.12 | 107.57±0.02 | 1.20±0.05 | 2.73±0.33 | 1.06±0.13 |
| **Jack** | 73.4±0.01 | 569.18±0.04 | 96.04±0.01 | 1.20±0.24 | 2.58±0.80 | 1.16±0.15 |
| **TL-1** | 97.14±0.00 | 743.67±0.01 | 77.74±0.00 | 0.84±0.00 | 2.92±0.11 | 1.04±0.02 |
| **W 82** | 80.62±0.01 | 665.46±0.05 | 96.97±0.00 | 1.19±0.05 | 2.35±0.05 | 0.78±0.02 |
| **Average** | 78.68 | 632.99 | 86.76 | 1.06 | 2.47 | 0.81 |

**Supplementary Table S9**. Mineral content of mature soybean on fresh weight (FW) basis

| **Cultivar** | **Magnesium (Mg) (mg/100 g)** | **Potassium (K) (mg/100 g)** | **Calcium (Ca)**  **(mg/100 g)** | **Manganese (Mn) (mg/100 g)** | **Iron (Fe) (mg/100 g)** | **Zinc (Zn) (mg/100 g)** |
| --- | --- | --- | --- | --- | --- | --- |
| **ZH 13** | 254.95±0.05 | 1852.46±0.44 | 188.23±0.05 | 2.86±0.69 | 7.29±1.34 | 3.02±0.40 |
| **ZH 35** | 241.09±0.06 | 2039.79±0.62 | 262.58±0.11 | 2.82±1.22 | 7.50±3.78 | 2.32±0.81 |
| **ZH 73** | 253.35±0.06 | 1959.17±0.28 | 212.38±0.04 | 3.37±0.88 | 7.07±1.71 | 2.69±0.29 |
| **ZH 78** | 243.52±0.07 | 1927.62±0.23 | 212.85±0.01 | 3.13±0.57 | 8.03±1.06 | 2.64±0.02 |
| **ZH 102** | 259.49±0.02 | 1955.47±0.09 | 250.96±0.02 | 2.69±0.58 | 9.25±1.22 | 2.77±0.19 |
| **ZH 106** | 244.32±0.02 | 1938.50±0.21 | 265.96±0.03 | 3.26±0.72 | 6.91±2.74 | 2.55±0.08 |
| **ZH 108** | 251.34±0.03 | 2148.08±0.11 | 260.84±0.00 | 2.76±0.10 | 8.08±1.06 | 2.34±0.31 |
| **ZH 111** | 252.05±0.06 | 2119.71±0.14 | 238.03±0.08 | 2.50±0.09 | 8.30±1.35 | 2.16±0.52 |
| **ZH 203** | 249.09±0.04 | 1948.32±0.38 | 285.73±0.06 | 3.22±0.15 | 7.06±1.05 | 3.30±0.40 |
| **Jack** | 276.12±0.03 | 1900.10±0.16 | 295.25±0.03 | 3.30±0.92 | 7.71±3.00 | 3.07±0.58 |
| **TL-1** | 245.86±0.05 | 2002.77±0.37 | 219.71±0.03 | 2.23±0.12 | 9.50±3.83 | 2.62±0.69 |
| **W 82** | 261.18±0.05 | 2049.71±0.24 | 271.26±0.02 | 3.36±0.25 | 7.62±0.27 | 2.63±0.08 |
| **Average** | 252.70 | 1986.81 | 246.98 | 2.96 | 7.86 | 2.68 |

**Supplementary Table S10.** Macronutrient content of Maodou on dry weight (DW) basis

| **Cultivar** | **Protein (g/100g)** | **Palmitic acid (g/100g)** | **Stearic acid （g/100g）** | **Oleic acid (g/100g)** | **Linoleic acid (g/100g)** | **Linolenic acid (g/100g)** | **Total fatty acid (g/100g)** | **Fructose (g/100g)** | **Glucose (g/100g)** | **Sucrose (g/100g)** | **Total soluble sugar (g/100g)** |
| --- | --- | --- | --- | --- | --- | --- | --- | --- | --- | --- | --- |
| **ZH 13** | 42.27±0.85 | 2.83±0.11 | 1.21±0.06 | 6.93±0.48 | 9.06±0.49 | 1.78±0.10 | 21.80±1.22 | 0.57±0.05 | 0.68±0.05 | 6.46±0.39 | 7.71±0.11 |
| **ZH 35** | 38.59±1.00 | 3.43±0.20 | 1.68±0.09 | 5.42±0.41 | 8.66±0.66 | 2.57±0.23 | 21.76±1.59 | 1.35±0.07 | 0.83±0.06 | 8.28±3.76 | 10.46±0.30 |
| **ZH 73** | 38.70±0.25 | 3.44±0.20 | 1.35±0.06 | 4.64±0.45 | 10.26±0.86 | 2.45±0.20 | 22.15±1.76 | 1.03±0.01 | 0.55±0.03 | 6.34±1.99 | 7.91±0.10 |
| **ZH 78** | 40.88±0.48 | 3.81±0.15 | 1.53±0.20 | 7.78±0.34 | 14.04±0.74 | 2.27±0.13 | 29.43±1.45 | 0.49±0.01 | 0.61±0.05 | 5.97±2.32 | 7.07±0.01 |
| **ZH 102** | 37.04±0.37 | 2.84±0.11 | 1.22±0.03 | 4.80±0.18 | 10.01±0.49 | 1.92±0.08 | 20.80±0.89 | 0.55±0.03 | 0.53±0.03 | 4.73±0.30 | 5.80±0.05 |
| **ZH 106** | 41.02±0.42 | 3.36±0.28 | 1.41±0.12 | 7.24±0.62 | 10.64±0.90 | 1.95±0.18 | 24.59±2.09 | 0.57±0.01 | 0.67±0.03 | 5.83±1.46 | 7.07±0.02 |
| **ZH 108** | 39.41±0.62 | 3.50±0.20 | 1.49±0.07 | 5.51±0.37 | 12.74±0.84 | 2.18±0.14 | 25.41±1.62 | 0.60±0.02 | 0.60±0.01 | 5.78±1.14 | 6.98±0.09 |
| **ZH 111** | 39.12±0.13 | 3.09±0.31 | 1.37±0.17 | 4.98±0.45 | 10.40±0.99 | 1.88±0.18 | 21.72±2.09 | 0.69±0.04 | 0.68±0.1 | 5.26±2.12 | 6.64±0.15 |
| **ZH 203** | 42.46±0.04 | 2.92±0.33 | 1.20±0.10 | 4.79±0.45 | 10.24±0.98 | 1.90±0.20 | 21.06±2.05 | 0.71±0.11 | 0.58±0.03 | 6.01±6.76 | 7.30±0.120 |
| **Jack** | 42.00±0.96 | 2.96±0.20 | 1.45±0.12 | 6.59±0.41 | 10.69±0.58 | 1.77±0.10 | 23.46±1.41 | 0.59±0.01 | 0.48±0.05 | 6.74±0.77 | 7.81±0.18 |
| **TL-1** | 41.27±0.17 | 3.26±0.06 | 1.26±0.03 | 6.12±0.19 | 10.69±0.45 | 2.14±0.11 | 23.47±0.78 | 0.35±0.04 | 0.74±0.00 | 6.26±3.49 | 7.34±0.18 |
| **W 82** | 40.57±0.41 | 3.12±0.13 | 1.48±0.06 | 6.31±0.25 | 11.27±0.41 | 2.01±0.07 | 24.18±0.91 | 0.72±0.02 | 0.52±0.03 | 5.89±3.79 | 7.13±0.03 |
| **Average** | 40.28 | 3.21 | 1.39 | 5.93 | 10.73 | 2.07 | 23.32 | 0.68 | 0.62 | 6.13 | 7.44 |

**Supplementary Table S11.** Macronutrient content of mature soybean on dry weight (DW) basis

| **Cultivar** | **Protein (g/100g)** | **Palmitic acid (g/100g)** | **Stearic acid （g/100g）** | **Oleic acid (g/100g)** | **Linoleic acid (g/100g)** | **Linolenic acid (g/100g)** | **Total fatty acid (g/100g)** | **Fructose (g/100g)** | **Glucose (g/100g)** | **Raffinose (g/100g)** | **Stachyose (g/100g)** | **Sucrose (g/100g)** | **Total soluble sugar(g/100g)** |
| --- | --- | --- | --- | --- | --- | --- | --- | --- | --- | --- | --- | --- | --- |
| **ZH 13** | 44.94±0.15 | 2.02±0.02 | 0.73±0.00 | 4.70±0.07 | 7.45±0.10 | 1.38±0.03 | 16.26±0.21 | 0.28±0.19 | 0.36±0.04 | 0.37±0.03 | 1.33±0.24 | 6.85±0.06 | 9.19±0.01 |
| **ZH 35** | 38.54±0.37 | 2.40±0.05 | 0.98±0.03 | 3.92±0.09 | 9.83±0.24 | 1.81±0.05 | 18.93±0.46 | 0.41±0.21 | 0.44±0.03 | 0.46±0.09 | 2.06±0.23 | 6.05±0.07 | 9.43±0.05 |
| **ZH 73** | 38.79±0.78 | 2.34±0.03 | 0.70±0.01 | 3.62±0.04 | 8.92±0.08 | 1.61±0.02 | 17.20±0.17 | 0.32±0.02 | 0.43±0.02 | 0.51±0.03 | 2.23±0.30 | 6.54±0.10 | 10.03±0.04 |
| **ZH 78** | 41.41±0.26 | 2.03±0.03 | 0.76±0.02 | 3.84±0.04 | 8.90±0.11 | 1.55±0.02 | 17.08±0.23 | 0.30±0.04 | 0.51±0.05 | 0.44±0.08 | 1.94±0.19 | 6.24±0.03 | 9.43±0.14 |
| **ZH 102** | 42.66±0.28 | 2.16±0.01 | 0.78±0.01 | 4.62±0.03 | 8.57±0.08 | 1.46±0.02 | 17.58±0.13 | 0.29±0.10 | 0.48±0.01 | 0.42±0.03 | 2.02±0.32 | 5.40±0.08 | 8.6±0.03 |
| **ZH 106** | 39.13±0.28 | 2.36±0.06 | 0.83±0.03 | 4.32±0.10 | 9.71±0.22 | 1.42±0.03 | 18.63±0.43 | 0.36±0.04 | 0.49±0.02 | 0.37±0.02 | 2.04±0.31 | 5.73±0.04 | 8.99±0.02 |
| **ZH 108** | 41.12±0.60 | 2.18±0.04 | 0.75±0.05 | 3.07±0.03 | 8.54±0.08 | 1.48±0.01 | 16.02±0.21 | 0.21±0.06 | 0.44±0.02 | 0.45±0.05 | 2.63±0.42 | 5.80±0.13 | 9.54±0.03 |
| **ZH 111** | 40.68±0.20 | 2.23±0.06 | 0.75±0.03 | 3.23±0.12 | 8.74±0.27 | 1.49±0.04 | 16.43±0.52 | 0.25±0.15 | 0.45±0.00 | 0.46±0.08 | 2.08±0.50 | 5.83±0.05 | 9.07±0.06 |
| **ZH 203** | 41.98±0.39 | 2.08±0.00 | 0.70±0.01 | 3.74±0.02 | 8.96±0.04 | 1.43±0.01 | 16.9±0.06 | 0.27±0.38 | 0.45±0.02 | 0.47±0.01 | 2.53±0.28 | 5.51±0.07 | 9.22±0.02 |
| **Jack** | 39.18±0.20 | 2.15±0.03 | 0.82±0.02 | 3.41±0.05 | 9.46±0.14 | 1.55±0.02 | 17.40±0.26 | 0.30±0.05 | 0.45±0.03 | 0.64±0.24 | 2.29±0.24 | 6.26±0.04 | 9.94±0.03 |
| **TL-1** | 41.70±0.37 | 2.03±0.01 | 0.70±0.01 | 4.12±0.02 | 7.38±0.01 | 1.52±0.00 | 15.75±0.05 | 0.19±0.15 | 0.37±0.00 | 0.47±0.03 | 2.46±0.17 | 5.25±0.06 | 8.74±0.05 |
| **W 82** | 40.63±0.24 | 2.08±0.03 | 0.81±0.01 | 3.50±0.05 | 8.87±0.13 | 1.43±0.02 | 16.70±0.23 | 0.32±0.05 | 0.41±0.01 | 0.44±0.09 | 1.80±0.69 | 6.07±0.06 | 9.03±0.08 |
| **Average** | 40.90 | 2.17 | 0.78 | 3.84 | 8.78 | 1.51 | 17.07 | 0.29 | 0.44 | 0.46 | 2.12 | 5.96 | 9.27 |

**Supplementary Table S12**. Micronutrient content of maodou on dry weight (DW) basis

| **Cultivar** | **THF (µg/ 100g)** | **5MTHF (µg/ 100g)** | **5,10MTHF (µg/ 100g)** | **10FFA (µg/ 100g)** | **5FTHF (µg/ 100g)** | **DHF (µg/ 100g)** | **FA (µg/ 100g)** | **Total folate (µg/ 100g)** | **Lutein (µg/100g)** | **Zeaxanthin (µg/100g)** | **β-cryptoxanthin (µg/100g)** | **α-carotene (µg/ 100g)** | **β-carotene (µg/100g)** | **Total carotenoid (µg/ 100g)** | **Delta tocopherol (µg/100g)** | **Gamma tocopherol (µg/100g)** | **Alpha tocopherol (µg/g100)** | **Total tocopherol (µg/100g)** |
| --- | --- | --- | --- | --- | --- | --- | --- | --- | --- | --- | --- | --- | --- | --- | --- | --- | --- | --- |
| **ZH 13** | 29.82±3.95 | 1425.05±56.67 | 31.16±0.26 | 9.06±0.72 | 179.82±10.08 | 9.98±2.01 | 3.67±1.15 | 1688.55±48.94 | 6089.04±204.32 | 176.1±6.93 | ND | 134.34±12.06 | 482±37.69 | 6881.49±2640.40 | 13122.63±603.38 | 28315.41±746.74 | 1505.66±76.64 | 42943.7±1349.65 |
| **ZH 35** | 56.61±9.08 | 2061.57±75.84 | 45.54±1.6 | 16.95±3.11 | 334.36±13.33 | 10.84±2.86 | 4.66±1.23 | 2530.53±92.85 | 19407.23±30.69 | 891.77±5.98 | 269.29±23.04 | 678.09±31.23 | 2406.78±115.14 | 23653.17±8244.19 | 7565.64±163.07 | 67695.24±1242.1 | 1764.17±382.73 | 77025.05±1164.22 |
| **ZH 73** | 46.87±2.12 | 1307.92±6.53 | 33.7±4.22 | 19.26±4.36 | 240.6±23.26 | 11.46±4.47 | 6.25±4.15 | 1666.06±37.46 | 12499.77±43.29 | 497.07±15 | 436.99±9.81 | 601.3±11.36 | 2906.82±61.17 | 16941.96±5198.26 | 10458.89±350.07 | 51222.77±180.02 | 1147.54±607.27 | 62829.21±649.69 |
| **ZH 78** | 35.78±4.55 | 1070.08±5.2 | 17.21±0.61 | 12.69±2.4 | 123.48±7.12 | 8.83±4.06 | 0.98±0.13 | 1269.06±10.27 | 8219.15±142.01 | 551.41±4.98 | 68.64±1.93 | 85.92±10.81 | 2053.28±41 | 10978.40±3463.43 | 10996.75±390.82 | 47661.32±1378.28 | 1113.47±617.48 | 59771.53±1742.05 |
| **ZH 102** | 33.14±6.25 | 766.72±39.77 | 25.45±2.95 | 13.05±2.66 | 205.59±4.72 | 10±1.25 | 2.07±1.18 | 1056.02±41.96 | 10651.98±130.95 | 330.79±0.46 | ND | 225.87±14.69 | 1316.51±16.28 | 12525.15±4582.09 | 9339.97±571.83 | 44296.04±474.44 | 1161.78±376.41 | 54797.79±755.09 |
| **ZH 106** | 37.99±3.03 | 959.22±28.06 | 41.48±3.77 | 11.65±1.19 | 314.44±25.52 | 9.58±1.53 | 2.71±1.52 | 1377.08±15.93 | 7648.26±11.75 | 453.88±2.92 | 492.04±13.81 | 27.62±10.36 | 436.08±21.62 | 9057.88±3268.25 | 11570.79±470.7 | 37488.2±1644.06 | 1050.14±49.37 | 50109.12±2155.63 |
| **ZH 108** | 36.31±5.46 | 768.52±25.18 | 25.21±6.35 | 10.97±4.17 | 184.6±16.75 | 9.41±5.56 | 1.88±2 | 1036.9±26.67 | 8401.73±105.59 | 359.91±6.01 | ND | 0±0.01 | 1264.52±13.34 | 10026.16±3612.92 | 9108.03±875.01 | 36071.74±1099.37 | 1465.43±210.75 | 46645.2±2054.92 |
| **ZH 111** | 38.5±4.85 | 945.62±15.85 | 28.32±5 | 11.88±3.09 | 275.41±14.42 | 6.67±1.83 | 2.78±0.96 | 1309.18±17.44 | 10368.63±13.56 | 360.34±17.26 | ND | 22.46±6.07 | 1062.19±57.23 | 11813.61±4495.96 | 11790.61±642.91 | 41234.78±2507.42 | 1594.85±98.98 | 54620.24±3247.5 |
| **ZH 203** | 34.84±5.21 | 720.96±24.58 | 28.17±1.21 | 11.6±3.87 | 220.07±5.28 | 8.75±2.21 | 2.86±0.17 | 1027.26±29.6 | 10296.22±54.9 | 454.8±19.4 | 686.2±14.54 | 228.09±9.74 | 1382.46±32.89 | 13047.76±4318.69 | 9468±340.7 | 45165.04±1510.98 | 1683.09±448.26 | 56316.13±2108.95 |
| **Jack** | 38.37±2.32 | 897.66±18.37 | 39.91±1.13 | 12.07±3.49 | 279.68±1.91 | 10.06±8.36 | 3.27±0.66 | 1281.02±28.2 | 11434.07±60.68 | 687.03±14.63 | 160.48±18.07 | 289.04±16.48 | 1304.07±73.84 | 13874.68±4861.03 | 9949.75±199.34 | 44483.25±965.63 | 1913.11±199.45 | 56346.1±958.66 |
| **TL-1** | 29.63±2.84 | 923.27±9.68 | 26.25±3.2 | 12.15±4.83 | 170.33±3.5 | 6.98±2.48 | 2.33±0.42 | 1170.94±4.64 | 3642.09±51.83 | 191.62±15.95 | ND | ND | 448.56±19.62 | 4282.26±1568.05 | 15780.75±301.54 | 24914.22±103.6 | 1168.13±64.88 | 41024.57±1573.49 |
| **W 82** | 38.91±6.6 | 1177.55±28.25 | 26.47±5.84 | 16.02±6.13 | 196.87±2.75 | 4.88±1.24 | 3.35±0.53 | 1464.05±23.3 | 9309.76±53.13 | 761.63±6.72 | 542.87±22.54 | 122.76±0.9 | 1749.43±19.23 | 12486.44±3854.86 | 11377.45±239.91 | 44026.39±1548.28 | 934.83±447.54 | 56338.68±1472.05 |
| **Average** | 38.06 | 1085.34 | 30.74 | 13.11 | 227.10 | 8.95 | 3.07 | 1406.39 | 9830.66 | 476.36 | 379.50 | 241.55 | 1401.06 | 12130.75 | 10877.44 | 42714.53 | 1375.18 | 54897.28 |

ND-not detected

**Supplementary Table S13.** Micronutrient content of mature soybean on dry weight (DW) basis

| **Cultivar** | **THF (µg/ 100g)** | **5MTHF (µg/ 100g)** | **5,10MTHF (µg/ 100g)** | **10FFA (µg/ 100g)** | **5FTHF (µg/ 100g)** | **DHF (µg/ 100g)** | **FA (µg/ 100g)** | **Total folate (µg/ 100g)** | **Lutein (µg/100g)** | **Zeaxanthin (µg/100g)** | **Total carotenoids (µg/100g)** | **Delta (µg/100g)** | **Gamma-tocopherol (µg/100g)** | **Alpha-tocopherol**  **(µg/g100)** | **Total tocopherol (µg/100g)** |  |
| --- | --- | --- | --- | --- | --- | --- | --- | --- | --- | --- | --- | --- | --- | --- | --- | --- |
| **ZH 13** | 53.88±1.77 | 198.64±1.71 | 6.59±1.07 | 22.44±2.54 | 131.17±9.41 | 2.6±0.91 | 22.16±1.38 | 437.5±9.00 | 1258.73±5.43 | ND | 1258.73±397.86 | 10186.25±147.42 | 14507.52±221.21 | 1049.67±29.73 | 25743.44±375.96 | |
| **ZH 35** | 59.94±5.31 | 196.71±4.38 | 6.45±0.72 | 20.24±3.37 | 117.86±1.07 | 3.49±1.81 | 23.73±0.54 | 428.44±8.77 | 1957.41±32.79 | 1.85±0.89 | 1959.27±617.82 | 10549.36±72.21 | 20092.75±194.07 | 1112.09±49.91 | 31754.21±296.24 | |
| **ZH 73** | 69.73±2.76 | 384.87±3.70 | 9.79±2.38 | 21.83±2.35 | 159.66±5.98 | 3.18±1.00 | 30.10±1.99 | 679.16±6.95 | 1837.68±23.97 | 14.68±2.60 | 1852.35±579.73 | 11374.56±224.17 | 17375.19±93.20 | 624.01±27.36 | 29373.76±280.53 | |
| **ZH 78** | 54.93±13.3 | 314.88±6.50 | 9.07±1.43 | 20.49±2.40 | 151.12±9.73 | 3.02±1.42 | 13.84±1.15 | 567.34±6.52 | 2250.56±46.75 | 89.25±0.41 | 2339.81±707.53 | 11040.01±134.95 | 18674.14±313.88 | 474.59±15.79 | 30188.75±432.09 | |
| **ZH 102** | 50.24±3.85 | 235.97±2.77 | 6.27±0.46 | 25.34±1.12 | 138.47±4.58 | 3.28±0.59 | 22.66±1.85 | 482.23±7.37 | 2538.59±38.73 | ND | 2538.59±801.51 | 7612.32±297.28 | 15231.21±162.54 | 868.28±50.57 | 23711.82±449.86 | |
| **ZH 106** | 51.83±5.83 | 216.05±1.88 | 7.40±0.54 | 17.76±0.65 | 128.69±3.82 | 3.42±1.5 | 22.73±0.46 | 447.87±2.73 | 1777.20±17.38 | 4.16±0.15 | 1781.36±561.26 | 9991.28±81.32 | 19432.55±132.18 | 983.31±56.81 | 30407.14±214.26 | |
| **ZH 108** | 59.95±1.17 | 225.27±0.79 | 9.25±0.93 | 24.29±1.84 | 152.23±0.12 | 3.40±1.92 | 32.59±6.13 | 506.98±7.50 | 1432.92±15.91 | ND | 1432.92±452.6 | 8040.09±115.72 | 16896.35±168.74 | 949.42±18.14 | 25885.86±249.05 | |
| **ZH 111** | 48.35±2.50 | 214.86±8.74 | 7.53±0.25 | 24.19±2.19 | 141.31±1.35 | 3.55±0.42 | 32.10±2.44 | 471.88±9.43 | 1427.50±28.67 | ND | 1427.50±450.5 | 9745.55±101.11 | 17297.9±154.25 | 907.84±31.06 | 27951.29±282.30 | |
| **ZH 203** | 50.38±1.25 | 233.82±2.45 | 7.88±0.26 | 20.65±1.41 | 133.18±1.97 | 2.41±1.16 | 18.46±1.91 | 466.77±2.27 | 2914.73±34.94 | 8.71±1.09 | 2923.44±920.21 | 7105.58±60.75 | 15745.55±292.87 | 994.07±20.28 | 23845.2±344.27 | |
| **Jack** | 53.09±1.55 | 183.95±2.30 | 6.91±0.30 | 21.88±1.45 | 109.58±4.51 | 3.86±0.43 | 29.11±3.91 | 408.38±7.73 | 2457.06±18.42 | ND | 2457.06±776.37 | 8040.19±44.72 | 20244.14±196.61 | 833.37±37.97 | 29117.69±279.24 | |
| **TL-1** | 44.67±2.60 | 255.96±0.73 | 8.50±0.67 | 37.37±1.16 | 198.66±6.20 | 2.48±0.54 | 27.73±3.21 | 575.39±7.02 | 325.37±4.82 | ND | 325.37±102.73 | 11724.21±325.52 | 15694.56±75.81 | 693.41±30.27 | 28112.17±359.29 | |
| **W 82** | 54.69±4.57 | 290.19±2.88 | 7.96±0.79 | 18.23±1.36 | 138.35±7.40 | 3.04±0.09 | 25.89±1.38 | 538.35±8.30 | 1787.27±50.41 | ND | 1787.27±563.64 | 9963.19±529.37 | 16973.87±403.54 | 681.79±41.95 | 27618.85±866.83 | |
| **Average** | 54.31 | 245.93 | 7.80 | 22.89 | 141.69 | 3.15 | 25.09 | 500.86 | 1830.42 | 23.73 | 1840.31 | 9614.38 | 17347.14 | 847.65 | 27809.18 |  |

ND- not detected

**Supplementary Table S14**. Mineral content of maodou on dry weight (DW) basis

| **Cultivar** | **Magnesium (Mg) mg/100 g** | **Potassium (K)**  **mg/100 g** | **Calcium (Ca)**  **mg/100 g** | **Manganese (Mn) mg/100 g** | **Iron (Fe)**  **mg/100 g** | **Zinc (Zn)**  **mg/100 g** |
| --- | --- | --- | --- | --- | --- | --- |
| **ZH 13** | 233.96±0.05 | 1893.71±0.44 | 199.25±0.05 | 2.99±0.69 | 6.11±1.34 | 3.27±0.40 |
| **ZH 35** | 203.79±0.06 | 1849.94±0.62 | 361.53±0.11 | 3.62±1.22 | 7.58±3.78 | 3.41±0.81 |
| **ZH 73** | 210.64±0.06 | 1825.56±0.28 | 266.72±0.04 | 3.72±0.88 | 6.37±1.71 | 2.94±0.29 |
| **ZH 78** | 267.69±0.07 | 1861.03±0.23 | 250.53±0.01 | 3.13±0.57 | 9.08±1.06 | 2.89±0.02 |
| **ZH 102** | 258.03±0.02 | 1911.42±0.09 | 279.82±0.02 | 3.09±0.58 | 7.27±1.22 | 1.97±0.19 |
| **ZH 106** | 208.88±0.02 | 1595.73±0.21 | 209.76±0.03 | 3.21±0.72 | 6.29±2.74 | 2.75±0.08 |
| **ZH 108** | 242.75±0.03 | 1984.04±0.11 | 212.05±0.00 | 2.74±0.10 | 7.19±1.06 | 2.24±0.31 |
| **ZH 111** | 251.68±0.06 | 2058.17±0.14 | 252.03±0.08 | 2.70±0.09 | 7.76±1.35 | 2.05±0.52 |
| **ZH 203** | 204.56±0.04 | 1958.63±0.38 | 321.17±0.06 | 3.59±0.15 | 8.16±1.05 | 3.18±0.40 |
| **Jack** | 224.80±0.03 | 1743.15±0.16 | 294.14±0.03 | 3.67±0.92 | 7.89±3.00 | 3.55±0.58 |
| **TL-1** | 256.95±0.05 | 1967.08±0.37 | 205.62±0.03 | 2.22±0.12 | 7.71±3.83 | 2.76±0.69 |
| **W 82** | 244.91±0.05 | 2021.45±0.24 | 294.55±0.02 | 3.61±0.25 | 7.13±0.27 | 2.38±0.08 |
| **Average** | 234.05 | 1889.16 | 262.26 | 3.19 | 7.38 | 2.78 |

**Supplementary Table S15.** Mineral content of mature soybean on dry weight (DW) basis

| **Cultivar** | **Magnesium (Mg) mg/100 g** | **Potassium (K)**  **mg/100 g** | **Calcium (Ca)**  **mg/100 g** | **Manganese (Mn) mg/100 g** | **Iron (Fe)**  **mg/100 g** | **Zinc (Zn)**  **mg/100 g** |
| --- | --- | --- | --- | --- | --- | --- |
| **ZH 13** | 272.36±0.05 | 1978.94±0.44 | 201.08±0.05 | 3.06±0.69 | 7.78±1.34 | 3.22±0.40 |
| **ZH 35** | 257.42±0.06 | 2178.00±0.62 | 280.37±0.11 | 3.01±1.22 | 8.01±3.78 | 2.47±0.81 |
| **ZH 73** | 271.04±0.06 | 2095.93±0.28 | 227.20±0.04 | 3.61±0.88 | 7.56±1.71 | 2.88±0.29 |
| **ZH 78** | 260.86±0.07 | 2064.83±0.23 | 228.00±0.01 | 3.35±0.57 | 8.60±1.06 | 2.83±0.02 |
| **ZH 102** | 279.17±0.02 | 2103.81±0.09 | 270.00±0.02 | 2.89±0.58 | 9.96±1.22 | 2.98±0.19 |
| **ZH 106** | 261.44±0.02 | 2074.33±0.21 | 284.59±0.03 | 3.48±0.72 | 7.39±2.74 | 2.73±0.08 |
| **ZH 108** | 269.27±0.03 | 2301.31±0.11 | 279.45±0.00 | 2.96±0.10 | 8.65±1.06 | 2.51±0.31 |
| **ZH 111** | 271.40±0.06 | 2282.45±0.14 | 256.30±0.08 | 2.69±0.09 | 8.93±1.35 | 2.33±0.52 |
| **ZH 203** | 267.00±0.04 | 2088.45±0.38 | 306.29±0.06 | 3.46±0.15 | 7.57±1.05 | 3.54±0.40 |
| **Jack** | 296.11±0.03 | 2037.67±0.16 | 316.62±0.03 | 3.54±0.92 | 8.27±3.00 | 3.30±0.58 |
| **TL-1** | 264.89±0.05 | 2157.83±0.37 | 236.72±0.03 | 2.40±0.12 | 10.24±3.83 | 2.83±0.69 |
| **W 82** | 279.59±0.05 | 2194.22±0.24 | 290.39±0.02 | 3.60±0.25 | 8.15±0.27 | 2.81±0.08 |
| **Average** | 270.88 | 2129.81 | 264.75 | 3.17 | 8.43 | 2.87 |

**Supplementary Table S16.** Isoflavone content of maodou on dry weight (DW) basis

| **Cultivar** | **Daidzin**  **µg/ g** | **Glycitin**  **µg/ g** | **Genistin**  **µg/ g** | **Malonyldaidzin**  **µg/g** | **Malonylglycitin**  **µg/ g** | **Malonylgenistin**  **µg/g** | **Total isoflavone**  **µg/ g** |
| --- | --- | --- | --- | --- | --- | --- | --- |
| **ZH 13** | ND | ND | 60.84±3.39 | 644.98±21.70 | 310.35±66.28 | 1566.31±36.10 | 2582.48±107.29 |
| **ZH 35** | ND | ND | ND | 130.22±4.14 | 269.94±0.87 | 76.78±0.24 | 476.94±3.03 |
| **ZH 73** | ND | ND | ND | 208.67±27.27 | 160.06±11.64 | 136.07±21.28 | 504.81±33.76 |
| **ZH 78** | ND | ND | ND | 102.65±13.07 | 245.57±23.24 | 290.56±12.25 | 638.78±31.99 |
| **ZH 102** | ND | ND | 5.00±1.13 | 342.10±19.43 | 393.26±10.99 | 528.73±1.92 | 1269.10±22.43 |
| **ZH 106** | ND | ND | ND | 116.61±27.53 | 234.77±44.63 | 319.51±12.52 | 670.88±79.21 |
| **ZH 108** | ND | 72.23±1.53 | 10.98±4 | 368.89±16.65 | 674.75±80.03 | 708.67±35.77 | 1835.53±135.24 |
| **ZH 111** | ND | 45.46±5.71 | 17.1±2.99 | 428.93±18.32 | 1082.45±7.40 | 590.54±6.78 | 2164.48±22.08 |
| **ZH 203** | ND | ND | ND | 233.60±13.16 | 429.79±15.00 | 352.47±17.8 | 1015.85±45.72 |
| **Jack** | ND | ND | ND | 202.89±21.21 | 476.65±35.64 | 203.36±14.32 | 882.90±65.21 |
| **TL-1** | 105.94±8.57 | 27.40±7.05 | 249.47±8.94 | 1718.56±59.65 | 971.65±5.98 | 3167.73±128.63 | 6240.74±193.64 |
| **W 82** | ND | 12.30±8.27 | ND | 389.79±12.32 | 524.30±13.57 | 393.77±5.58 | 1320.15±39.74 |
| **Average** | 105.94 | 39.34 | 68.68 | 407.32 | 481.13 | 694.54 | 1633.55 |

ND- not detected

**Supplementary Table S17.** Isoflavone content of mature soybean on dry weight (DW) basis

| **Cultivar** | **Daidzin**  **µg/ g** | **Glycitin**  **µg/ g** | **Genistin**  **µg/ g** | **Malonyldaidzin**  **µg/g** | **Malonylglycitin**  **µg/ g** | **Malonylgenistin µg/g** | **Total isoflavone**  **µg/ g** |
| --- | --- | --- | --- | --- | --- | --- | --- |
| **ZH 13** | 65.17±5.76 | 42.69±5.81 | 104.83±0.34 | 852.80±11.14 | 423.94±9.99 | 1585.95±15.02 | 3075.37±14.76 |
| **ZH 35** | 53.09±2.35 | 50.54±2.51 | 135.81±4.41 | 940.59±2.47 | 537.70±25.38 | 2011.08±17.94 | 3728.81±13.45 |
| **ZH 73** | 108.01±3.91 | 22.32±0.32 | 170.09±3.53 | 1840.00±6.10 | 546.03±56.73 | 2621.71±5.78 | 5308.15±64.51 |
| **ZH 78** | 61.13±2.18 | 52.86±4.19 | 179.33±4.59 | 895.78±17.56 | 394.37±15.00 | 2329.27±1.77 | 3912.75±25.61 |
| **ZH 102** | 77.75±2.40 | 30.41±2.72 | 121.49±1.01 | 1336.73±7.59 | 436.17±5.57 | 1701.72±6.86 | 3704.27±10.66 |
| **ZH 106** | 35.92±2.25 | 37.19±4.35 | 78.09±4.43 | 708.90±7.93 | 360.70±30.97 | 1449.28±27.77 | 2670.08±60.00 |
| **ZH 108** | 32.74±1.38 | 35.61±1.05 | 48.92±2.40 | 824.07±11.88 | 639.79±30.21 | 1267.09±1.96 | 2848.23±47.60 |
| **ZH 111** | 60.91±2.16 | 28.53±2.05 | 87.28±3.32 | 1197.60±5.82 | 497.09±0.95 | 1925.81±27.11 | 3797.22±34.72 |
| **ZH 203** | 118.31±2.84 | 33.39±3.38 | 182.48±2.30 | 1673.94±1.62 | 477.69±20.57 | 2223.79±3.04 | 4709.60±22.52 |
| **Jack** | 59.55±0.40 | 38.10±5.66 | 115.18±0.70 | 981.64±8.03 | 512.20±16.23 | 1915.76±3.27 | 3622.45±29.94 |
| **TL-1** | 82.17±4.46 | 34.37±1.58 | 170.60±6.50 | 1210.12±8.99 | 533.45±10.52 | 2192.71±20.88 | 4223.42±23.32 |
| **W 82** | 115.75±2.17 | 40.48±12.48 | 151.56±1.70 | 1568.36±11.82 | 495.23±30.83 | 2130.05±4.50 | 4501.42±44.81 |
| **Average** | 72.54 | 37.21 | 128.81 | 1169.21 | 487.87 | 1946.18 | 3841.81 |

**Supplementary Table S18.** Descriptive analysis of quality traits analysed in this study based on dry weight basis

| **Trait (DW)** | **Stage** | **Minimum** | **Maximum** | **Mean** | **SD** | **CV (%)** | **Pvalue** |
| --- | --- | --- | --- | --- | --- | --- | --- |
| Protein (g/100 g) | Maodou | 37.04 | 42.46 | 40.28 | 1.70 | 4.21 | 0.4018013 |
|  | Mature soybean | 38.54 | 44.94 | 40.90 | 1.85 | 4.53 |  |
| Palmitic acid (g/100 g) | Maodou | 2.83 | 3.81 | 3.21 | 0.30 | 9.48 | 2.7671E-10 |
|  | Mature soybean | 2.02 | 2.40 | 2.17 | 0.14 | 6.25 |  |
| Stearic acid (g/100 g) | Maodou | 1.20 | 1.68 | 1.39 | 0.15 | 10.65 | 1.5564E-11 |
|  | Mature soybean | 0.70 | 0.98 | 0.78 | 0.08 | 10.34 |  |
| Oleic acid (g/100 g) | Maodou | 4.64 | 7.78 | 5.93 | 1.06 | 17.85 | 3.5939E-06 |
|  | Mature soybean | 3.07 | 4.70 | 3.84 | 0.52 | 13.54 |  |
| Linoleic acid (g/100 g) | Maodou | 8.66 | 14.04 | 10.73 | 1.46 | 13.61 | 0.00047417 |
|  | Mature soybean | 7.38 | 9.83 | 8.78 | 0.76 | 8.65 |  |
| Linolenic acid (g/100 g) | Maodou | 1.77 | 2.57 | 2.07 | 0.26 | 12.45 | 6.9566E-07 |
|  | Mature soybean | 1.38 | 1.81 | 1.51 | 0.11 | 7.60 |  |
| Total fatty acid (g/100 g) | Maodou | 20.80 | 29.43 | 23.32 | 2.42 | 10.37 | 3.1608E-08 |
|  | Mature soybean | 15.75 | 18.93 | 17.07 | 0.97 | 5.69 |  |
| Fructose (g/100 g) | Maodou | 0.35 | 1.35 | 0.68 | 0.26 | 38.74 | 5.1087E-05 |
|  | Mature soybean | 0.19 | 0.41 | 0.29 | 0.06 | 20.30 |  |
| Glucose (g/100 g) | Maodou | 0.48 | 0.83 | 0.62 | 0.10 | 16.46 | 1.1747E-05 |
|  | Mature soybean | 0.36 | 0.51 | 0.44 | 0.04 | 9.91 |  |
| Raffinose (g/100 g) | Maodou | ND | ND | ND | ND | ND | NA |
|  | Mature soybean | 0.37 | 0.64 | 0.46 | 0.07 | 15.33 |  |
| Stachyose (g/100 g) | Maodou | ND | ND | ND | ND | ND | NA |
|  | Mature soybean | 1.33 | 2.63 | 2.12 | 0.35 | 16.70 |  |
| Sucrose (g/100 g) | Maodou | 4.73 | 8.28 | 6.13 | 0.86 | 14.07 | 0.55425087 |
|  | Mature soybean | 5.25 | 6.85 | 5.96 | 0.47 | 7.89 |  |
| Total soluble sugar (g/100 g) | Maodou | 5.80 | 10.46 | 7.44 | 1.11 | 14.89 | 2.343E-05 |
|  | Mature soybean | 8.60 | 10.03 | 9.27 | 0.43 | 4.66 |  |
| THF (µg/ 100g) | Maodou | 29.63 | 56.61 | 38.06 | 7.41 | 19.48 | 1.0029E-05 |
|  | Mature soybean | 44.67 | 69.73 | 54.31 | 6.54 | 12.04 |  |
| 5MTHF (µg/ 100g) | Maodou | 720.96 | 2061.57 | 1085.34 | 376.38 | 34.68 | 1.2696E-07 |
|  | Mature soybean | 183.95 | 384.87 | 245.93 | 58.11 | 23.63 |  |
| 5,10MTHF (µg/ 100g) | Maodou | 17.21 | 45.54 | 30.74 | 8.09 | 26.31 | 2.0017E-09 |
|  | Mature soybean | 6.27 | 9.79 | 7.80 | 1.16 | 14.90 |  |
| 10FFA (µg/ 100g) | Maodou | 9.06 | 19.26 | 13.11 | 2.86 | 21.83 | 8.1307E-06 |
|  | Mature soybean | 17.76 | 37.37 | 22.89 | 5.11 | 22.34 |  |
| 5FTHF (µg/ 100g) | Maodou | 123.48 | 334.36 | 227.10 | 63.13 | 27.80 | 0.00022319 |
|  | Mature soybean | 109.58 | 198.66 | 141.69 | 22.85 | 16.12 |  |
| DHF (µg/ 100g) | Maodou | 4.88 | 11.46 | 8.95 | 1.90 | 21.18 | 6.7715E-10 |
|  | Mature soybean | 2.41 | 3.86 | 3.15 | 0.45 | 14.39 |  |
| FA (µg/ 100g) | Maodou | 0.98 | 6.25 | 3.07 | 1.37 | 44.75 | 6.24E-12 |
|  | Mature soybean | 13.84 | 32.59 | 25.09 | 5.61 | 22.37 |  |
| Total folate (µg/ 100g) | Maodou | 1027.26 | 2530.53 | 1406.39 | 417.50 | 29.69 | 2.1604E-07 |
|  | Mature soybean | 408.38 | 679.16 | 500.86 | 77.56 | 15.49 |  |
| B-carotene (µg/100 g) | Maodou | 436.08 | 2906.82 | 1401.06 | 778.05 | 55.53 | NA |
|  | Mature soybean | ND | ND | ND | ND | ND |  |
| Lutein (µg/100 g) | Maodou | 3642.09 | 19407.23 | 9830.66 | 3867.79 | 39.34 | 4.4525E-07 |
|  | Mature soybean | 325.37 | 2914.73 | 1830.42 | 686.21 | 37.49 |  |
| Zeaxanthin (µg/100 g) | Maodou | 176.10 | 891.77 | 476.36 | 218.42 | 45.85 | 2.3473E-07 |
|  | Mature soybean | 1.85 | 89.25 | 23.73 | 36.95 | 155.70 |  |
| B-cryptoxanthin (µg/100 g) | Maodou | 68.64 | 686.20 | 379.50 | 221.16 | 58.28 | NA |
|  | Mature soybean | ND | ND | ND | ND | ND |  |
| A-carotene (µg/100g) | Maodou | 22.46 | 678.09 | 241.55 | 227.61 | 94.23 | NA |
|  | Mature soybean | ND | ND | ND | ND | ND |  |
| Total carotenoid (µg/100g) | Maodou | 4282.26 | 23653.17 | 12130.75 | 4903.91 | 40.43 | 3.2576E-07 |
|  | Mature soybean | 325.37 | 2923.44 | 1840.31 | 692.88 | 37.65 |  |
| Delta tocopherol (µg/100 g) | Maodou | 7565.64 | 15780.75 | 10877.44 | 2135.14 | 19.63 | 0.11121492 |
|  | Mature soybean | 7105.58 | 11724.21 | 9614.38 | 1546.93 | 16.09 |  |
| Gamma tocopherol (µg/100 g) | Maodou | 24914.22 | 67695.24 | 42714.53 | 11004.42 | 25.76 | 7.7798E-08 |
|  | Mature soybean | 14507.52 | 20244.14 | 17347.14 | 1912.24 | 11.02 |  |
| Alpha tocopherol (µg/100 g) | Maodou | 934.83 | 1913.11 | 1375.18 | 318.69 | 23.17 | 6.5313E-05 |
|  | Mature soybean | 474.59 | 1112.09 | 847.65 | 192.04 | 22.66 |  |
| Total tocopherol (µg/100 g) | Maodou | 41024.57 | 77025.05 | 54897.28 | 9577.21 | 17.45 | 3.266E-09 |
|  | Mature soybean | 23711.82 | 31754.21 | 27809.18 | 2571.72 | 9.25 |  |
| Magnesium (Mg) (mg/100 g) | Maodou | 203.79 | 267.69 | 234.05 | 22.94 | 9.80 | 4.5761E-05 |
|  | Mature soybean | 257.42 | 296.11 | 270.88 | 10.48 | 3.87 |  |
| Potassium (K) (mg/100 g) | Maodou | 1595.73 | 2058.17 | 1889.16 | 128.02 | 6.78 | 3.2225E-05 |
|  | Mature soybean | 1978.94 | 2301.31 | 2129.81 | 96.32 | 4.52 |  |
| Calcium (Ca) (mg/100 g) | Maodou | 199.25 | 361.53 | 262.26 | 50.82 | 19.38 | 0.89047785 |
|  | Mature soybean | 201.08 | 316.62 | 264.75 | 35.20 | 13.29 |  |
| Manganese (Mn) (mg/100 g) | Maodou | 2.22 | 3.72 | 3.19 | 0.47 | 14.84 | 0.90610574 |
|  | Mature soybean | 2.40 | 3.61 | 3.17 | 0.39 | 12.44 |  |
| Iron (Fe) (mg/100 g) | Maodou | 6.11 | 9.08 | 7.38 | 0.85 | 11.56 | 0.00826813 |
|  | Mature soybean | 7.39 | 10.24 | 8.43 | 0.91 | 10.85 |  |
| Zinc (Zn) (mg/100 g) | Maodou | 1.97 | 3.55 | 2.78 | 0.53 | 18.96 | 0.64661757 |
|  | Mature soybean | 2.33 | 3.54 | 2.87 | 0.35 | 12.36 |  |
| Daidzin (µg/g) | Maodou | 105.94 | 105.94 | 105.94 | NA | NA | 2.861E-05 |
|  | Mature soybean | 32.74 | 118.31 | 72.54 | 28.81 | 39.72 |  |
| Glycitin (µg/g) | Maodou | 12.30 | 72.23 | 39.34 | 25.77 | 65.51 | 0.00311484 |
|  | Mature soybean | 28.53 | 52.86 | 37.21 | 4.91 | 13.20 |  |
| Genistin (µg/g) | Maodou | 5.00 | 249.47 | 68.68 | 103.43 | 150.60 | 0.00043382 |
|  | Mature soybean | 48.92 | 182.48 | 128.81 | 44.78 | 34.77 |  |
| Malonyldaidzin (µg/g) | Maodou | 102.65 | 1718.56 | 407.32 | 441.89 | 108.49 | 0.00014255 |
|  | Mature soybean | 708.90 | 1840.00 | 1169.21 | 367.78 | 31.46 |  |
| Malonylglycitin (µg/g) | Maodou | 160.06 | 1082.45 | 481.13 | 293.10 | 60.92 | 0.93926195 |
|  | Mature soybean | 360.70 | 639.79 | 487.87 | 76.00 | 15.58 |  |
| Malonylgenistin (µg/g) | Maodou | 76.78 | 3167.73 | 694.54 | 871.85 | 125.53 | 0.00016189 |
|  | Mature soybean | 1267.09 | 2621.71 | 1946.18 | 390.56 | 20.07 |  |
| Total Isoflavone (µg/g) | Maodou | 476.94 | 6240.74 | 1633.55 | 1599.82 | 97.93 | 0.00028383 |
|  | Mature soybean | 2670.08 | 5308.15 | 3841.81 | 769.79 | 20.04 |  |

SD - standard deviation; CV- coefficient of variation

**Supplementary Table S19.** Expression of biosynthesis genes during seed development

| **Trait** | **Enzyme** | **Gene** | **Expression during seed development** |
| --- | --- | --- | --- |
| Tocopherol | HPPD | *Glyma.02G284600* | Increases with maturity |
|  | HPT | *Glyma.13G097800* | Decreases with maturity |
|  | HPT | *Glyma.09G103500* | Decreases with maturity |
|  | HPT | *Glyma.02G168000* | Decreases with maturity |
| Carotenoid | Phytoene synthase | *Glyma.02G240200* | Decreases with maturity |
|  | Phytoene synthase | *Glyma.14G031200* | Decreases with maturity |
|  | Phytoene synthase | *Glyma.14G209700* | Decreases with maturity |
| Folate | GTPCHI | *Glyma.08G347400* | Decreases with maturity |
|  | ADCS | *Glyma.20G179400* | Decreases with maturity |
|  | FPGS | *Glyma.18G034200* | Decreases with maturity |
|  | GGH | *Glyma.13G267800* | Decreases with maturity |
| Isoflavone | IFS | *Glyma.07G202300* | Increases with maturity |
